# CC16 drives VLA-2-dependent SPLUNC1 expression

**DOI:** 10.3389/fimmu.2023.1277582

**Published:** 2023-11-20

**Authors:** Natalie Iannuzo, Holly Welfley, Nicholas C. Li, Michael D. L. Johnson, Joselyn Rojas-Quintero, Francesca Polverino, Stefano Guerra, Xingnan Li, Darren A. Cusanovich, Paul R. Langlais, Julie G. Ledford

**Affiliations:** ^1^Department of Cellular and Molecular Medicine, University of Arizona, Tucson, AZ, United States; ^2^Asthma and Airway Disease Research Center, Tucson, AZ, United States; ^3^BASIS Tucson North, Tucson, AZ, United States; ^4^Department of Immunobiology, University of Arizona, Tucson, AZ, United States; ^5^Baylor College of Medicine, Houston, TX, United States; ^6^Department of Medicine, Division of Pulmonary, Allergy, Critical Care, and Sleep Medicine, University of Arizona, Tucson, AZ, United States; ^7^Department of Medicine, Division of Genetics, Genomics, and Precision Medicine, University of Arizona, Tucson, AZ, United States; ^8^Department of Medicine, Division of Endocrinology, University of Arizona, Tucson, AZ, United States

**Keywords:** CC16, mycoplasma pneumoniae, SPLUNC1, airway epithelia, mass spectrometry

## Abstract

**Rationale:**

CC16 (Club Cell Secretory Protein) is a protein produced by club cells and other non-ciliated epithelial cells within the lungs. CC16 has been shown to protect against the development of obstructive lung diseases and attenuate pulmonary pathogen burden. Despite recent advances in understanding CC16 effects in circulation, the biological mechanisms of CC16 in pulmonary epithelial responses have not been elucidated.

**Objectives:**

We sought to determine if CC16 deficiency impairs epithelial-driven host responses and identify novel receptors expressed within the pulmonary epithelium through which CC16 imparts activity.

**Methods:**

We utilized mass spectrometry and quantitative proteomics to investigate how CC16 deficiency impacts apically secreted pulmonary epithelial proteins. Mouse tracheal epithelial cells (MTECS), human nasal epithelial cells (HNECs) and mice were studied in naïve conditions and after Mp challenge.

**Measurements and main results:**

We identified 8 antimicrobial proteins significantly decreased by CC16^-/-^ MTECS, 6 of which were validated by mRNA expression in Severe Asthma Research Program (SARP) cohorts. Short Palate Lung and Nasal Epithelial Clone 1 (SPLUNC1) was the most differentially expressed protein (66-fold) and was the focus of this study. Using a combination of MTECs and HNECs, we found that CC16 enhances pulmonary epithelial-driven SPLUNC1 expression via signaling through the receptor complex Very Late Antigen-2 (VLA-2) and that rCC16 given to mice enhances pulmonary SPLUNC1 production and decreases *Mycoplasma pneumoniae* (Mp) burden. Likewise, rSPLUNC1 results in decreased Mp burden in mice lacking CC16 mice. The VLA-2 integrin binding site within rCC16 is necessary for induction of SPLUNC1 and the reduction in Mp burden.

**Conclusion:**

Our findings demonstrate a novel role for CC16 in epithelial-driven host defense by up-regulating antimicrobials and define a novel epithelial receptor for CC16, VLA-2, through which signaling is necessary for enhanced SPLUNC1 production.

## Introduction

Club cell secretory protein (CC16; also known as CC10, CCSP, and uteroglobin) is a homodimeric pneumoprotein encoded by the *SCGB1A1* gene and produced by club cells and non-ciliated epithelial cells in the distal pulmonary epithelium (1). Despite its origin in the lungs, CC16 is also detectable in the serum, making it a potential biomarker for lung epithelial integrity and lung function (1–3). Although the complete biological functions of CC16 have not been elucidated, studies have shown that this protein has anti-inflammatory and antioxidant properties, which may protect against the development of obstructive lung disease (4–6). Additionally, our group has previously shown that CC16 deficiency leads to dramatically altered pulmonary function and enhanced airway remodeling (7) and that, by binding to the integrin Very Late Antigen-4 (VLA-4) on leukocytes, CC16 can reduce leukocyte adhesion to endothelial cells, lung infiltration, and airway inflammation (8).

We have also previously shown that during infection with *Mycoplasma pneumoniae* (Mp), CC16 deficient (CC16^-/-^) mice had significantly higher Mp burden compared to WT mice. This increased Mp burden appeared to be driven by an epithelial impairment since mouse tracheal epithelial cells (MTECs) deficient in CC16 also had significantly higher Mp colonization, compared to wildtype (WT) cells (8, 9). These findings supported the possibility that increased Mp burden observed in the CC16^-/-^ mice and MTECs may be due to decreased pulmonary epithelial-driven antimicrobial responses (9). A better understanding of how CC16 mediates epithelial-driven antimicrobial responses and how this regulation aids in the resolution of pulmonary infections is of paramount importance given that several patient populations have been described as having low CC16 levels, including asthma, chronic obstructive pulmonary disease (COPD) and cystic fibrosis patients (10–14). Therefore, in this study, we used mass spectrometry and quantitative proteomics and identified 8 apically secreted antimicrobial proteins that are significantly downregulated within the pulmonary epithelium when CC16 is absent. We found that of those 8 identified from MTECS, 6 were validated as significantly associated with CC16 mRNA expression in epithelial cells from asthma patients from 2 SARP cohorts (cross sectional and longitudinal) (15). These proteins include *BPIFA1* (encodes Short Palate Lung and Nasal Epithelial Clone 1; SPLUNC1), *TF* (encodes Transferrin), *LTF* (encodes Lactotransferrin), *SCGB3A2* (encodes Secretoglobin 3A2*), SFTPD* (encodes Surfactant Protein-D), and *LYZ* (encodes Lyzosyme).

SPLUNC1 was the most differentially expressed antimicrobial protein, with very high expression from WT MTECs and very low to non-detectable expression from CC16^-/-^ MTECs and became the focus of our mechanistic studies. Since SPLUNC1 was the most differentially expressed protein from MTECs dependent on CC16 expression, along with the known roles of SPLUNC1 in respiratory host defense (16–21), we set up a series to additional experiments to understand if and how CC16 regulates the expression of SPLUNC1 using WT and CC16^-/-^ MTECs and mice, as well as human nasal epithelial cells (HNECs) and clinical data from the SARP cohorts.

## Methods

### Experimental mice

All experiments were handled in accordance with University of Arizona on IACUC approved animal protocols. WT and CC16^-/-^ male mice on a C57BL/6J background were obtained at ~6-8 weeks of age at the time of rCC16 treatment, or males and females >12 weeks of age for MTECs as more cells are recovered for growth on Transwells from aged mice. All mice were born and raised in the same room in the University of Arizona Health Sciences animal facility and were tested to be specific-pathogen free according to standard protocols using sentinel mice from the same room.

### Mouse tracheal epithelial cell isolation

MTECs were isolated as previously described (9). Detailed MTEC isolation methods can be found in the supplement.

### MTEC culture media, supplements, and *in vitro* culturing

To reduce biological variation by decreasing the number of mice used for MTECs, we followed a recent culturing protocol published by Eenjes et al. (22). Detailed MTEC culturing methods can be found in the supplement.

### NCI-H292 cell culturing

NCI-H292 cells (human pulmonary mucoepidermoid carcinoma cells) (ATCC; Manassas, VA) were cultured in T75 flasks using ATCC-formulated RPMI-1640 medium (ATCC 30-2001) supplemented with 10% FBS, 100 U/mL penicillin and 100 μg/mL streptomycin at 37°C, 5% CO_2_. Cell culture medium was replaced every 48 hrs. Once 90% confluency was reached, the cells were removed from by flask by incubation (37°C, 5% CO_2_) with 0.25% trypsin-EDTA for 10 min. Cells were passaged (1:8 dilution) into fresh RPMI-1640 medium supplemented with 10% FBS.

### Human nasal epithelial cell isolation and culturing

Human nasal epithelial cell (HNEC) brushings were obtained from healthy donors under University of Arizona IRB-approved protocols (PI: Ledford). After collection, HNECs were submerged in sputolysin (MilliporeSigma; Burlington, MA) diluted in RPMI supplemented with an antibiotic/antimycotic (Gibco; Waltham, MA) for 30 min (37°C, 5% CO_2_) followed by centrifugation (350 RCF, 5 min, 4°C). The HNECs were washed three times using 10% FBS/RPMI followed by centrifugation (350 RCF, 5 min, 4°C). HNECs were seeded in collagen coated T25 flasks at a density of 5x10^5^ cells per 25 cm^2^ using PneumaCult Ex-Plus media (StemCell Technologies; Vancouver, CA). HNECs were removed from the T25 flasks by incubation (37°C, 5% CO_2_) with ACF enzymatic dissociation solution for 7-8 min, followed by addition of ACF enzyme inhibition solution to stop the reaction. Cells were then plated onto Costar Transwell membranes (12 mm, 0.4 μm membrane pores) at a density of 89,600 cells per membrane. After the initial seeding period, PneumaCult Basal ALI media (StemCell Technologies; Vancouver, VA) supplemented with PneumaCult Basal Media (StemCell Technologies; Vancouver, CA) was replaced every other day. When the cells reached 80% confluency (~5 days), the culture medium was replaced daily, only on the basolateral side, to establish an ALI. After the first day in an ALI, the medium was replaced every 48 hrs. Once full confluency was obtained, the cells were maintained at an ALI for 14 days.

### rCC16 treatment for mice

WT and CC16^-/-^ mice were treated with rCC16 (16.7 *μ*g/mouse) intravenously for 3 days as described previously (8). rCC16 was made by Dr. Michael Johnson as previously described (8) and diluted in sterile, USP-grade 0.9% NaCl prior to administration.

### rCC16 and D67A treatment for MTECs

WT and CC16^-/-^ MTECs were treated with WT rCC16 (25 *μ*g/mL) for 12-, 24-, and 48-hrs, and D67A rCC16 (25 *μ*g/mL) for 24 hrs (8). WT and D67A rCC16, diluted in MTEC differentiation media, was added to the apical and basolateral chambers of the MTEC cultures.

### Mp infection for WT MTECs

Mp infection in MTECs was performed as previously described (9). In short, after the apical surface of the WT MTECs was washed with sterile 1x PBS, Mp (1x10^6^ Mp/200 *μ*L inoculum) was added to the apical side of the MTECs and incubated for 24 hrs (37°C, 5% CO_2_).

### In-solution tryptic digestion

In-solution tryptic digestion of the apically secreted proteins from WT and CC16^-/-^ MTECs was performed as described (23). Detailed tryptic digestion methods can be found in the **Supplement**.

### Mass spectrometry and data search

HPLC-ESI-MS/MS was performed as previously described (24) in positive ion mode on a Thermo Scientific Orbitrap Fusion Lumos tribrid mass spectrometer fitted with an EASY-Spray Source (Thermo Scientific, San Jose, CA). Detailed mass spectrometry and data search methods can be found in the supplement.

### Label-free quantitative proteomics

Progenesis QI for proteomics software (version 2.4, Nonlinear Dynamics Ltd., Newcastle upon Tyne, UK) was used to perform ion-intensity based label-free quantification as previously described (25). Detailed proteomics methods can be found in the supplement.

### SYBR green real-time PCR for measurement of gene expression in mice, MTECs, and HNECs

Gene expression for all experiments was performed as previously described (9). Detailed RT-PCR methods can be found in the supplement.

### Measuring *BPIFA1* in mice and MTECs, and HNECs

*BPIFA1* (forward: 5’-GTCCACCCTTGCCACTGAACCA-3’; reverse: 5’-CACCGCTGAGAGCATCTGTGAA-3’) was examined in lung tissue from CC16^-/-^ mice treated with saline (control) or rCC16, as well as WT and CC16^-/-^ MTECs treated with media (control), rCC16 (25 *μ*g/mL), or VLA-2 inhibitor BTT 3033 IC_50_ = 130 nM) (R&D Systems; Minneapolis, MN) by RT-PCR using a Sybr-green compatible system. *GAPDH* (forward: 5’-CCTGCACCACCAACTGCTTA-3’; reverse: 5’-GTCTTCTGGGTGGCAGTGAT-3’) was used as a housekeeping control for these sets of experiments.

### Measuring Mp burden in WT MTECs

Measuring Mp burden by RT-PCR was performed as previously described (9). In short, the *MP-SPECIFIC P1 ADHESIN* gene was used to assess Mp burden in infected WT MTECs (forward: 5′-CGCCGCAAAGAT GAATGAC-3′; reverse: 5′-TGTCCTTCCCCATCTAACAGTTC-3′) using a Sybr-green compatible system. *GAPDH* was used as a housekeeping control for these sets of experiments. Non-infected MTECs were used as negative controls.

### Determination of SPLUNC1 and integrin protein levels by western blotting

To determine SPLUNC1 protein levels in the lungs, right lung tissue from each sample was homogenized with 400 *μ*L of RIPA (radioimmunoprecipitation assay) buffer (Teknova; Hollister, CA) with protease inhibitors (Roche; Basel, Switzerland). After homogenization, the lungs were centrifuged at 12,000 rpm for 10 min at 4°C, and the supernatant was collected. Protein concentrations from lung lysates and apical secretions were quantified using a Pierce BCA Protein Assay Kit (Thermo Fisher Scientific; Waltham, MA). Equal amounts of lysate and apical secretion were loaded onto Mini-Protean TGX precast gels (Bio-Rad Laboratories; Hercules, CA). To determine SPLUNC1 protein levels in bronchoalveolar lavage fluid (BALF) from mice, equal volumes of BALF were loaded into Mini-Protean TGX precast gels, respectively. To determine integrin protein levels in MTECs, a standard concentration (10 *μ*g/mL) was loaded into each well of a Mini-Protean TGX precast gel. Antibodies for SPLUNC1 (Proteintech; Rosemont, IL), ITGA2 (Cell Signaling; Danvers, MA), ITGA4 (Cell Signaling; Danvers, MA), ITGB1 (Cell Signaling; Danvers, MA), and GAPDH (Cell Signaling; Danvers, MA) were used according to the manufacturer’s recommendations. All primary antibodies were diluted 1:1000 in 5% (w/v) nonfat dry milk-1X TBST and required an anti-rabbit secondary antibody (Cell Signaling; Danvers, MA). The secondary antibody was diluted 1:2000 in 5% (w/v) nonfat dry milk-1X TBST. A ChemiDoc imaging system and Image Lab software (Thermo Fisher Scientific; Waltham, MA) were used to image and quantify the densitometry of each western blot.

### Human tracheal epithelium single-cell RNA sequencing data analysis

Single-cell RNA sequencing data of epithelial cells isolated from six healthy adult tracheal donors described in Goldfarbmuren et al. were used to assess the expression level and cell-type specificity of genes of interest (26). Detailed methods on this analysis can be found in the supplement.

### Flow cytometry on WT and CC16^-/-^ mouse lungs and MTECs

Detailed flow cytometry methods can be found in the supplement.

### VLA-2 inhibition in WT MTECs and healthy HNECs

Differentiated WT MTECs and healthy HNECs were pre-treated apically with BTT 3033 at its respective IC_50_ concentration for 30 minutes (37°C; 5% CO_2_). After pre-treatment with the BTT3033, media or rCC16 (25 *μ*g/mL) was apically added to their respective cells. Treated MTECs and HNECs were incubated for 24 hrs (37°C; 5% CO_2_).

### rSPLUNC1 Treatment for CC16^-/-^ mice

CC16^-/-^ mice were infected with Mp intranasally (1x10^8^ Mp/mL; 50 μL/mouse) for 2 hrs, followed by oropharyngeal treatment with rSPLUNC1 (50 μg/mL; 40 μL/mouse) (R&D Systems; Minneapolis, MN). Mice were given a rSPLUNC1 (10 μg/mL; 40 μL/mouse) 24 hrs after the initial treatment. rSPLUNC1 was diluted in USP-grade 0.9% NaCl prior to administration.

### *In-silico* prediction of CC16 binding with integrin α2 subunit

Using the PRISM^®^ software that predicts protein-protein interactions at a structural level (27), we assessed the protein-protein interaction between rat-CC16, integrin subunit α2, and the cytoplasmic tail of β1, using available protein 3D structure models in the Protein Data Bank (PDB, https://www.rcsb.org). Please note that there is no available human CC16 structure available in the PDB to conduct the *in-silico* test. The protein interactions are measured by the Fiberdock energy scoring (28, 29), which measures the negative energy required for such interactions. The negative scores obtained reflect the spontaneity of the binding. The higher the negative number the more spontaneous the protein-protein binding.

### Protein affinity calculations using ISLAND

To determine binding affinity between CC16 and VLA-2, a sequence-based protein binding affinity predictor called ISLAND was used (30). The human protein sequences for CC16 and the α2 subunit were inputted and predicted binding and protein affinities were obtained using the ΔΔG and Kd values.

### Severe asthma research program participants

SARP is a currently active NHLBI-sponsored multicenter program involving non-smokers with mild to severe asthma and a subset of healthy controls. Participants in SARP were comprehensively phenotyped using standard protocols as described previously (31, 32). Bronchoscopy was performed on a subset of participants in the SARP longitudinal cohort (n = 156) and cross-sectional cohort (n = 155) to obtain epithelial cells from brush biopsies for RNAseq and microarray mRNA chip analysis, respectively (**Supplementary Table 1**). The RNAseq and microarray expression data have been deposited and can be accessed through dbGaP (phs001446) and GEO (GSE63142 and GSE43696), respectively (15, 33). SARP was approved by the appropriate institutional review board including informed consent.

### Meta-analysis of genes correlated with CC16 mRNA expression levels

Correlation analyses of CC16 mRNA expression levels in human bronchial epithelial cells with other candidate genes (*BPIFA1*, *BPIFB1*, *TF*, *LTF*, *SCGB3A2*, *SFTPB*, *SFTPD*, *LYZ*, *ITGA2*, and *ITGB1*) in the SARP cross-sectional and longitudinal cohorts were performed using Spearman correlation as described previously (15, 33). Meta-analysis of Spearman correlation coefficient (rho) was performed in the SARP cohorts (n = 311) using metacor R package based on the Olkin-Pratt fixed-effect meta-analytical model (34) as described previously (15).

### Statistical analysis

For all mouse and MTEC experiments, statistics were analyzed using Prism 8 (GraphPad). For all analysis of mouse and MTEC experiments, significance was determined by either Unpaired *t* test or One-way ANOVA for multiple comparisons, as appropriate.

## Results

### CC16 deficiency results in decreased apical antimicrobial expression by pulmonary epithelial cells

**Figure 1:**
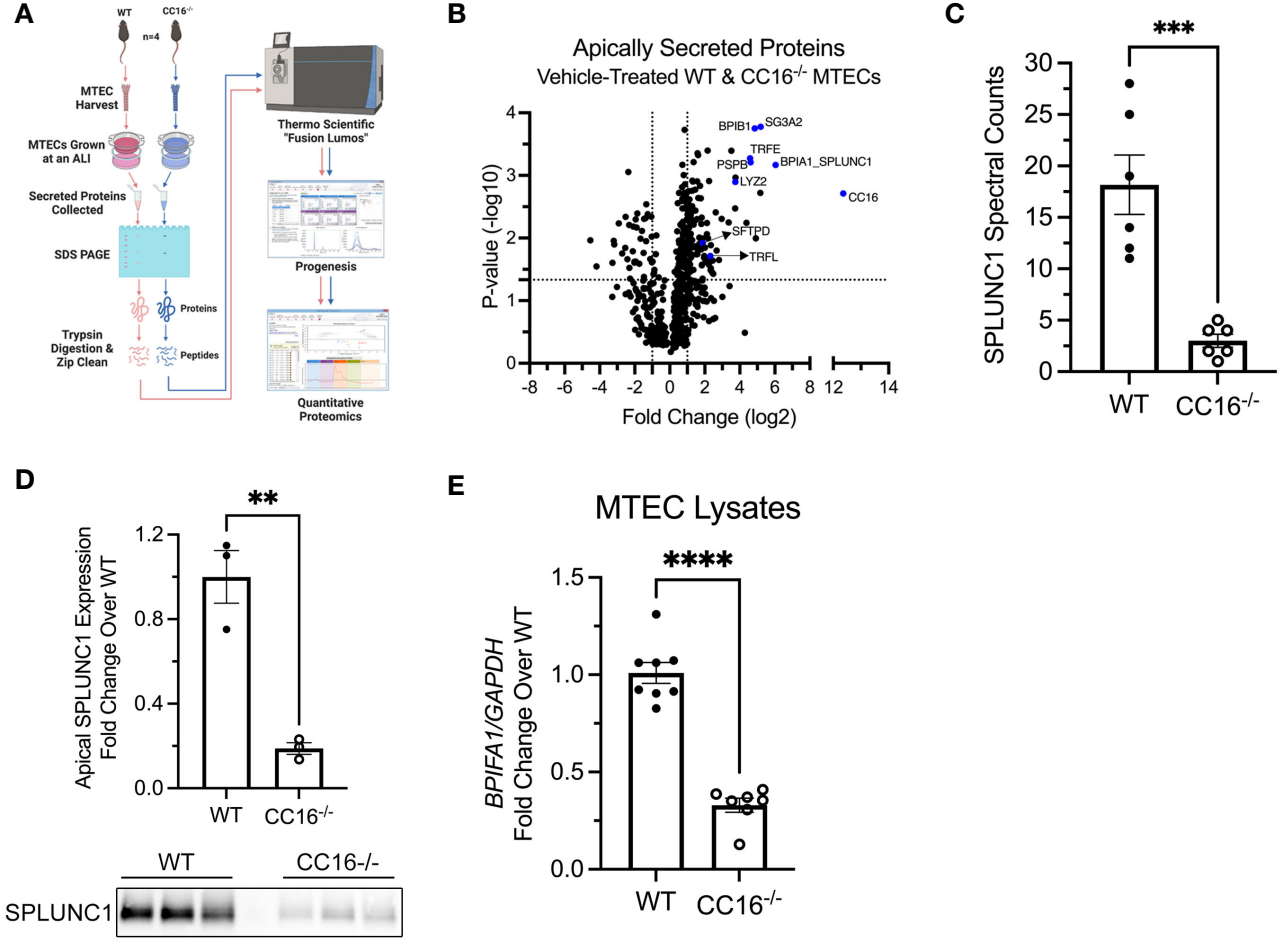
Apical expression of SPLUNC1 is decreased in CC16^-/-^ MTECs. **(A)** Schematic of the experimental design for the mass spectrometry and quantitative proteomics approach to identify apically secreted proteins by WT and CC16^-/-^ MTECs. **(B)** Volcano plot illustrating the apically secreted proteins identified. Above the horizontal black line represents the cut-off for a *p* value of <0.05, while the two vertical lines represent the cut-off values of 2-fold change in either the positive or negative direction. Selected antimicrobial proteins are highlighted in red and labeled. **(C)** Spectral counts of SPLUNC1 protein secreted by WT and CC16^-/-^ MTECs by LC-MS/MS. ^**^^*^P<0.001 Unpaired *t* test. Data are presented as mean ± SEM from six independent experiments. **(D)** SPLUNC1 apical protein expression was confirmed for control-treated WT and CC16^-/-^ MTECs by western blotting. The same volume of protein (20*μ*l) was loaded for each sample. ^**^P<0.01 Unpaired *t* test. Data are presented as mean±SEM. **(E)** WT (n=8) and CC16^-/-^ (n=7) MTECs were treated with media (control) for 48 hours, after which *BPIFA1* expression was measured by RT-PCR with *GAPDH* as a housekeeping control. ^****^P<0.0001 Unpaired *t* test. Data are presented as mean±SEM.

Apically secreted proteins from vehicle/control (media) treated WT and CC16^-/-^ MTECs were identified and characterized using mass spectrometry and quantitative proteomics, respectively (**Figure 1A**). CC16 and SPLUNC1 (boxed in the volcano plot) were the most differentially expressed proteins detected by mass spectrometry from the WT MTECs in comparison to the CC16^-/-^ MTECs (**Figure 1B**). More specifically, CC16 and SPLUNC1 secretion by the WT MTECs was approximately 5000 and 66 times greater, respectively, compared to CC16^-/-^ MTECs (**Table 1**). In total, we found 8 antimicrobial associated proteins that were significantly higher in apical secretions from WT MTECs that are CC16 sufficient (colored blue and labeled in the volcano plot) compared to MTECs deficient in CC16.

**Table 1:** Select apically secreted antimicrobial proteins whose expression is decreased in CC16^-/-^ MTECs.

Protein Abbreviation	Protein Name	Average Fold Change Relative to CC16^-/-^ MTECs	Average P-value
CC16	Club Cell Secretory Protein	5267.1555	0.001940074
BPIA1_SPLUNC1	BPI Fold-Containing Family A Member 1; Short-Palate Lung and Nasal Epithelial Clone 1	66.1294	0.000679336
SCGB3A2	Secretoglobin Family 3A Member 2	36.5174	0.00016662
BPIB1	BPI Fold-Containing Family B Member 1	28.8412	0.000177805
PSPB	Pulmonary Surfactant Protein B	24.4547	0.000615467
TRFE	Transferrin	23.9729	0.00053206
LYZ2	Lysozyme C-2	13.4078	0.001273525
TRFL	Lactotransferrin	4.9948	0.019350103
SFTPD	Pulmonary Surfactant Protein D	3.6991	0.011988244

SPLUNC1 spectral counts, which is a measurement of protein abundance, were significantly higher in the apical secretions from WT MTECs, compared to CC16^-/-^ MTECs (**Figure 1C**). Protein levels were confirmed by western blotting of SPLUNC1 in the apical secretions taken from the ALI cultures (**Figure 1D**). Additionally, *BPIFA1* gene expression by RT-PCR was higher in WT MTECs, compared to CC16^-/-^ MTECs (**Figure 1E**), suggesting that CC16 may regulate *BPIFA1* expression at the gene level.

### Recombinant CC16 increases SPLUNC1 expression in WT and CC16^-/-^ MTECs and mice

**Figure 2:**
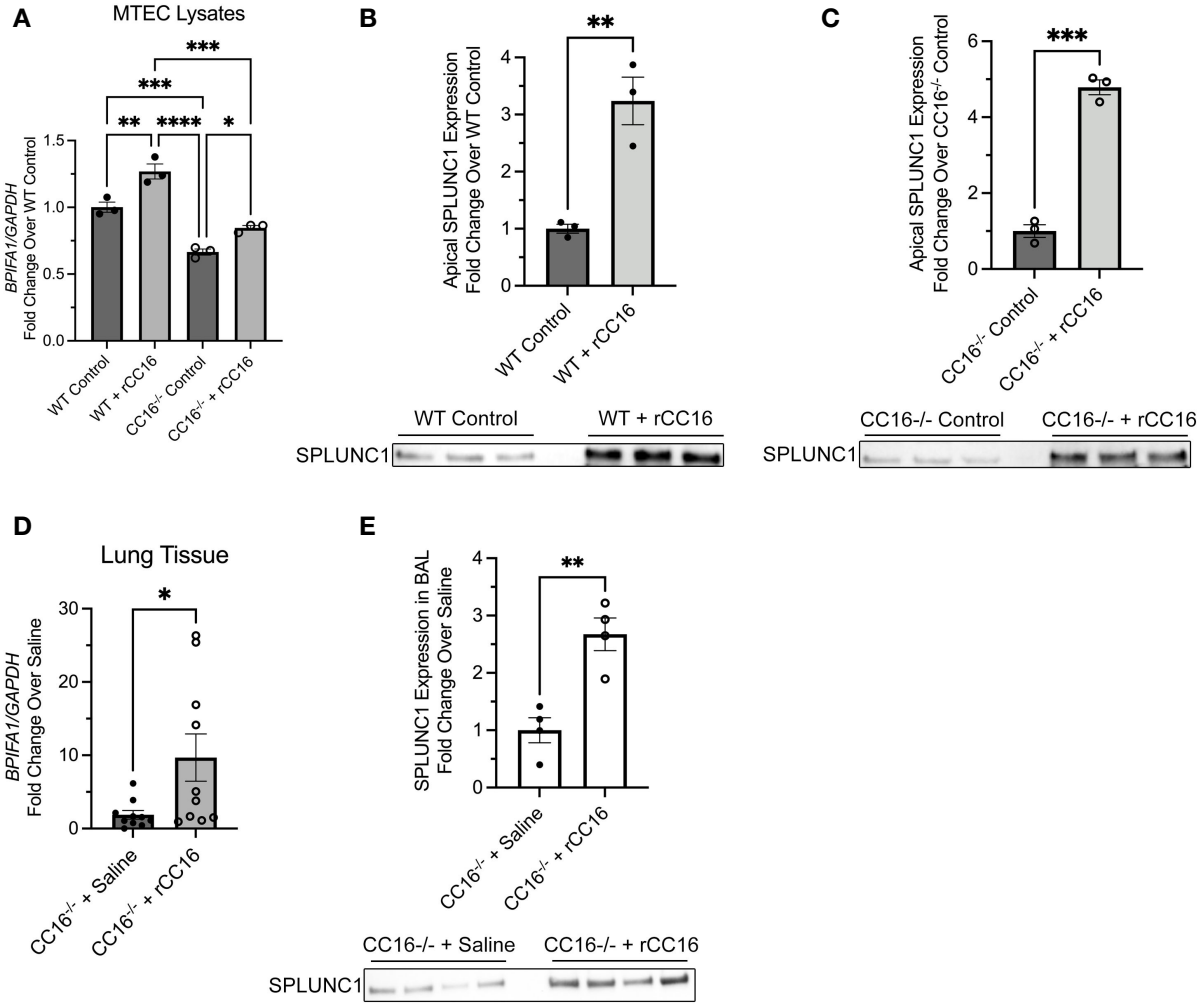
rCC16 treatment increases SPLUNC1 expression *in vitro* and *in vivo*. **(A)** WT (n=3) and CC16^-/-^ (n=3) MTECs were treated with media (control) or rCC16 (25*μ*g/ml) for 24 hours, after which *BPIFA1* expression in cell lysates was measured by RT-PCR with *GAPDH* as a housekeeping control. ^*^P<0.05, ^*^^*^P<0.01, ^*^^**^P<0.001, ^*^^***^P<0.0001 by One-Way ANOVA Tukey’s multiple comparison test. SPLUNC1 apical protein expression was confirmed for control- and rCC16-treated WT **(B)** and CC16^-/-^ **(C)** MTECs by western blotting (n=3 per group). The same volume of protein (20*μ*l) was loaded for each sample. ^**^P<0.01, ^***^P<0.001 by *Unpaired t* test. Data are presented as mean±SEM. **(D)** CC16**^-/-^** mice were treated intravenously with saline (n=10) and rCC16 (n=10) for 3 days, after which *BPIFA1* expression in lung tissue was measured by RT-PCR with *GAPDH* as a housekeeping control. ^*^P<0.05 by Unpaired *t* test. **(E)** SPLUNC1 protein expression was measured in the BALF from the 3-day saline (n=4) and rCC16 (n=4) treated CC16^-/-^ mice. The same volume of protein (20*μ*l) was loaded for each sample. ^**^P<0.01 by Unpaired *t* test. Data are presented as mean±SEM.

We next sought to determine if exogenous rCC16 could augment SPLUNC1 expression at the gene and protein level. For this, WT and CC16^-/-^ MTECs were incubated with media (control) or recombinant CC16 (rCC16) for 12-, 24-, and 48-hrs to determine which time point resulted in the most significant upregulation of SPLUNC1 expression. The 24-hr time point resulted in the highest *BPIFA1* gene expression by RT-PCR in both WT and CC16^-/-^ MTECs and was therefore chosen as the time point for assessment moving forward (**Figure 2A**). Apical expression of SPLUNC1 protein levels in WT (**Figure 2B**) and CC16^-/-^ (**Figure 2C**) MTECs by western blotting confirmed that rCC16 treatment significantly increases SPLUNC1 expression. However, we did not observe significantly increased SPLUNC1 expression between WT and CC16^-/-^ MTEC lysates (**Supplementary Figure 1A**).

Additional studies were carried out in CC16^-/-^ mice treated intravenously with saline or rCC16 for 3 days, followed by assessment of *BPIFA1* gene expression in lung tissue and SPLUNC1 protein levels in the bronchoalveolar lavage fluid (BALF). rCC16 treatment resulted in significantly increased *BPIFA1* gene expression in the lung tissue of CC16^-/-^ mice, compared to saline-treated CC16^-/-^ mice (**Figure 2D**). SPLUNC1 protein levels, assessed by western blotting of BALF, showed that CC16^-/-^ mice treated with rCC16 had significantly higher SPLUNC1 levels in the luminal lining fluid of their lungs, but not in their lung tissue (**Supplementary Figure 1B**), compared to saline treated mice (**Figure 2E**).

### SCGB1A1 and antimicrobial gene expression significantly and positively associated in human cohorts

We next chose to examine if *SCGB1A1* and *BPIFA1* associate in human samples using the well-established SARP cohorts. Correlation analysis between *SCGB1A1* and *BPIFA1* mRNA expression was performed using data from two human cohorts – the cross-sectional cohort (SARP1-2) and the longitudinal cohort (SARP3). Data included mRNA expression from asthma and non-asthma participants. The cross-sectional (n=155; p=0.017) data and the longitudinal (n=156; p=0.021) data showed significant correlation between *SCGB1A1* and *BPIFA1* bronchial gene expression with meta-analysis significance of p=2.9x10^-4^ (rho=0.19) (**Table 2**). Next, the same set of 8 identified proteins from apical secretions of MTECS (**Table 1**) were assessed for corresponding gene expression in the human SARP cohort data. Of the 8 genes assessed by meta-analysis, 6 were found to be positively and significantly correlated with CC16 expression, including: *BPIFA1, TF, LTF*, *SCGB3A2, SFTPD*, and *LYZ.* This human data provides additional support and is in line with the findings from MTECS that respiratory epithelial expression of CC16 is associated with gene expression of several key antimicrobial genes.

**Table 2:** Correlation of mRNA expression levels of eight antimicrobial genes with CC16 in bronchial epithelial cells in SARP.

Mouse Protein	Human Gene	Cross-Sectional Cohort (n = 155)		Longitudinal Cohort (n = 156)		Meta-Analysis*	
		rho	*P* value	rho	*P* value	rho	*P* value
CC16	*SCGB1A1*	–	–	–	–	–	–
BPIA1_SPLUNC1	*BPIFA1*	0.19	0.017	0.19	0.021	0.19	2.9x10^-4^
BPIB1	*BPIFB1*	-0.071	0.38	-0.14	0.073	-0.11	0.027
TRFE	*TF*	0.028	0.73	0.24	0.003	0.13	0.009
TRFL	*LTF*	0.51	1.3x10^-11^	0.55	<2.2x10^-16^	0.53	1.6x10^-39^
SCGB3A2	*SCGB3A2*	0.23	0.003	0.018	0.82	0.13	0.012
PSPB	*SFTPB*	-0.027	0.74	0.022	0.78	-0.002	0.48
SFTPD	*SFTPD*	0.19	0.019	0.034	0.67	0.11	0.024
LYZ2	*LYZ*	-0.062	0.44	-0.17	0.034	-0.12	0.019

*Meta-analysis of Spearman correlation coefficient (rho) was performed in the SARP cohorts (n=311) using metacor R package on the Olkin-Pratt fixed-effect meta-analytical model.

### SPLUNC1 is highly expressed by mucus and submucosal gland secretory cells in the pulmonary epithelium

**Figure 3:**
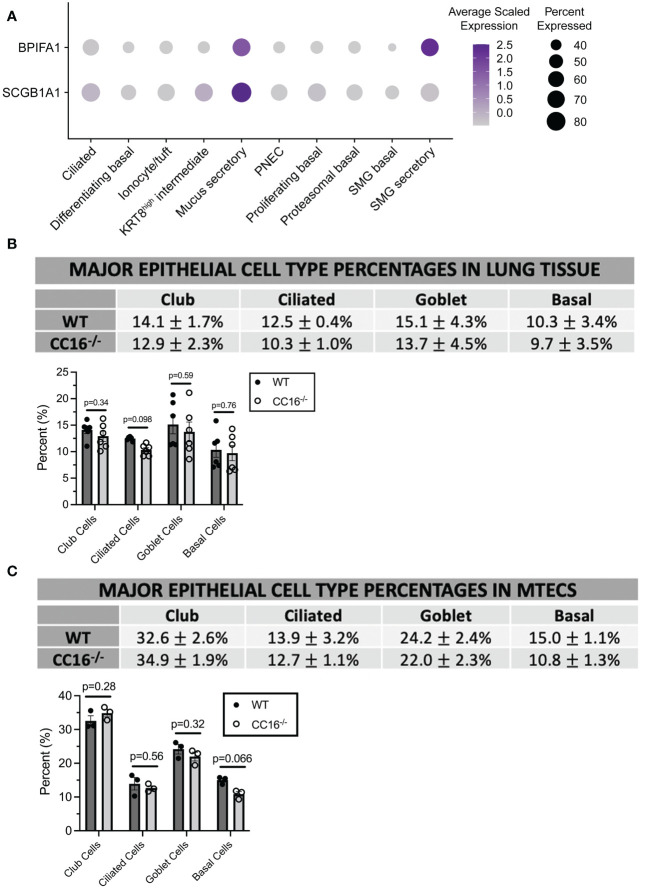
CC16 deficiency does not affect epithelial cell differentiation. **(A)** Dot plot showing the average normalized expression of *BPIFA1* and *SCGB1A1* (rows) across cell types (columns) generated from single-cell RNA sequencing data of healthy adult tracheal epithelial cells described in Goldfarburen et al. (33). The color of the dot represents the average gene expression in the cell type and the diameter of the dot indicates the fraction of cells that express the gene within the cell type. **(B, C)** Major epithelial cell type expression was measured in WT (n=6) and CC16^-/-^ (n=6) mice **(B)** and in WT (n=3) and CC16^-/-^ (n=3) MTECS **(C)** using flow cytometry. Indirect FACs staining was used to identify club (CYP2F2), ciliated (TUBA1A), goblet (MUC5AC), and basal (KRT5) expression. No significant differences in epithelial cell expression were observed between WT and CC16^-/-^ mice and MTECs by Unpaired *t* test. NS=not significant. Data are presented as mean±SEM.

There has been some discrepancy between publications in identifying which respiratory cell types produce SPLUNC1 (17, 18, 35–37). To better understand the source of *BPIFA1* expression, we utilized published single-cell RNA sequencing (scRNA-seq) data from healthy adult tracheal epithelial cells described in Goldfarbmuren et al. (26) (**Figure 3A**). *BPIFA1* and *SCGB1A1* were expressed (expression greater than 0) in 47.4% and 68.6% of the total cells in the dataset, respectively. However, the cell type annotated as “mucus secretory” cells by the original authors, which includes the club cell population, exhibited significant upregulation of both *BPIFA1* (average log fold-change = 4.58 relative to all other cells, adjusted p-value = 2.4x10^-6^) and *SCGB1A1* (average log fold-change = 4.46, adjusted p-value = 7.7x10^-6^). “Submucosal gland secretory” cells also had significant upregulation of *BPIFA1* (average log fold-change = 3.98, adjusted p-value = 3.5x10^-5^), while *SCGB1A1* expression was not significantly upregulated in this population. Furthermore, considering individual mucus secretory cells, we found a subtle but significant positive relationship between *BPIFA1* and *SCGB1A1* expression (Pearson correlation coefficient = 0.33, p-value = 9.7 x10^-31^) (**Supplementary Figure 2**). Taken together, these data suggest that mucus secretory cells are the primary source of *BPIFA1* expression in the airways.

### CC16 deficiency does not affect major epithelial cell populations in mice and MTECs

To confirm that differences in major epithelial cell populations were not driving the differences in SPLUNC1 expression, flow cytometry was performed on WT and CC16^-/-^ mice and on MTECs grown at an ALI for two weeks for major epithelial cell markers (club, ciliated, goblet, and basal cells). In WT and CC16^-/-^ mouse lungs (**Figure 3B**, **Supplementary Figure 3**) and MTECs (**Figure 3C**, **Supplementary Figure 4**), there were no significant differences in the expression of these epithelial cell populations.

### CC16 does not activate the SPLUNC1 promoter

To mechanistically understand how CC16 activates *BPIFA1* expression, we used a luciferase reporter assay in which NCI-H292 cells were transfected with the *BPIFA1* promoter, treated with rCC16 for 24 hrs, and quantified promoter activation by luminescence. To elucidate how CC16 regulates SPLUNC1 expression, we sought to determine if CC16 directly activates the SPLUNC1 promoter. Additionally, we aimed to determine if there are apically secreted protein(s) or intracellular protein(s) from WT and/or CC16^-/-^ MTECs that activate the *BPIFA1* promoter, and if this activation is dependent in CC16. Overall, transfection of the reporter constructs into the NCI-H292 cells was successful, as *ACTB* luminescence was significantly higher than the negative control and SPLUNC1 luminescence. However, rCC16 did not activate the *BPIFA1* promoter directly (**Supplementary Figure 5A**). Additionally, there were no apically secreted proteins (**Supplementary Figure 5B**), nor intracellular proteins (**Supplementary Figure 5C**), from the WT and CC16^-/-^ MTECs that significantly activated the *BPIFA1* promoter. These results suggest CC16-mediated induction of SPLUNC1 expression is not direct through enhancing promoter activity, but instead likely involves intermediate signaling.

### CC16 does not signal through TLR2 to activate SPLUNC1 expression in MTECs

Multiple papers have shown that TLR2 activation stimulates SPLUNC1 expression in airway epithelial cells (17, 38, 39); therefore, we sought to determine if CC16 signals through TLR2 to activate SPLUNC1 expression in WT MTECs. WT MTECs were incubated with a TLR2 blocking antibody (*α*-TLR2 antibody) or IgG1 isotype control antibody, followed by incubation with media (negative control), rCC16, or Pam3CSK4 (positive control). For the *α*-TLR2 antibody-treated MTECs, *BPIFA1* gene expression was significantly increased during rCC16 treatment, compared to Pam3CSK4 treatment, indicating that CC16 does not signal through TLR2 to activate *BPIFA1* expression (**Supplementary Figure 6A**). If CC16 were to signal through TLR2, *BPIFA1* expression levels would be at similar levels as the Pam3CSK4-treated MTECs, since Pam3CSK4 activates TLR2, and this interaction would be disrupted due to *α*-TLR2 antibody incubation. Apical expression of SPLUNC1 further indicated that CC16 does not signal through TLR2 in WT MTECs (**Supplementary Figure 6B**).

### VLA-2 is necessary for rCC16-induced SPLUNC1

**Figure 4:**
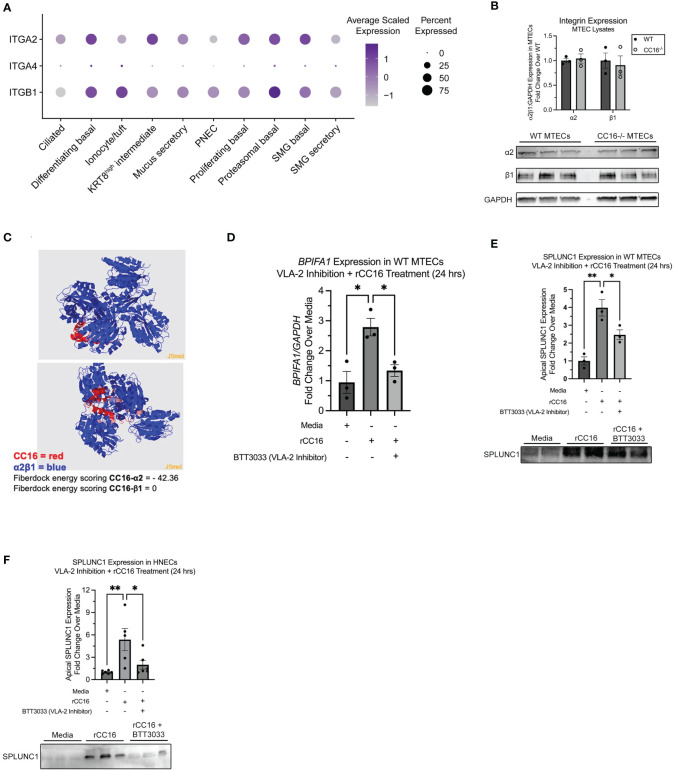
Inhibition of VLA-2 decreases SPLUNC1 expression in WT MTECs and healthy HNECs. **(A)** Dot plot of *α*2, *β*1, and *α*4 integrin subunit (gene names: *ITGA2*, *ITGA4*, and *ITGB1*, respectively) expression across healthy human adult tracheal epithelial cells from single-cell RNA sequencing data as described in Goldfarbmuren et al. **(B)** Expression of *α*2 and *β*1 integrin subunits was verified in WT MTECs (n=3) and CC16^-/-^ MTECs (n=3) by western blotting. The same amount of protein (10 *μ*g) was loaded for each sample. *α*2 and *β*1 protein levels are normalized to GAPDH. **(C)** Protein-protein interactions between CC16 and the integrin receptor components *α*2*β*1 using protein 3D structure modeling in the Protein Data Bank. The strength of interactions were measured by the Fiberdock energy scoring. The negative scores reflect the strength of binding. **(D)** WT MTECs (n=3) were treated with media, rCC16 (25 *μ*g/mL), or rCC16 in combination with BTT3033 (VLA-2 inhibitor) (130 nM) for 24 hrs. Following the treatments, *BPIFA1* gene expression was measured by RT-PCR with *GAPDH* as a housekeeping control. ^*^P<0.05 by One-Way ANOVA Tukey’s multiple comparison test. **(E)** SPLUNC1 apical protein expression was assessed in the WT MTECs treated with media, rCC16, and rCC16 in combination with BTT3033 by western blotting (n=3/group). The same volume of protein (20*μ*l) was loaded for each sample. ^*^P<0.05, ^**^P<0.01 by One-Way ANOVA Tukey’s multiple comparison test. **(F)** SPLUNC1 apical protein expression was assessed by western blotting in healthy human nasal epithelial cells (HNECs) treated with media, rCC16 (25 *μ*g/mL), or rCC16 in combination with BTT3033 (VLA-2 inhibitor) (130 nM) for 24 hrs (n=5-6 per group). ^*^P<0.05, ^**^P<0.01 by One-Way ANOVA Tukey’s multiple comparison test.

Our group has previously shown that CC16 binds to VLA-4 to prevent leukocyte extravasation into the lung (8). Using scRNA-seq data from healthy human tracheal epithelial cells, we confirmed that VLA-4 is expressed in the airways, albeit at very low levels. However, we found that VLA-2 (*α*2*β*1) is highly expressed across numerous airway cell types (**Figure 4A**). Based on this, we aimed to determine if CC16 could interact with VLA-2 to influence SPLUNC1 expression within the pulmonary epithelium. First, we confirmed that the integrins alpha-2 (*α*2) and beta-1 (*β*1) were expressed in both WT and CC16^-/-^ MTECs at similar levels by western blotting and the doublet bands appeared as expected (**Figure 4B**). Next, a VLA-2 specific inhibitor, BTT3033, was added to WT MTECs in the presence of rCC16 for 24 hrs, followed by assessment of SPLUNC1 gene and apical protein expression by RT-PCR and western blotting, respectively. Compared to the positive control (rCC16), rCC16 was unable to upregulate SPLUNC1 when cells were co-treated with the BTT3033 inhibitor and had levels like the media only negative control (**Figures 4D, E**). For translatability, similar experiments were conducted in healthy primary HNECs, which produced similar results demonstrating that rCC16 could not upregulate SPLUNC1 when VLA-2 was inhibited (**Figure 4F**).

From the SARP data, human bronchial epithelial cells mRNA expression levels of alpha 2 subunit of VLA-2 (gene name: *ITGA2*) were positively and significantly correlated with CC16, further supporting the potential role of VLA-2 in CC16-SPLUNC1 signaling pathway (**Table 3**). Along these lines, we assessed the protein-protein interaction between CC16 and the integrin receptor components *α*2*β*1. The strength of protein-protein interactions were measured by Fiberdock energy scoring. CC16 and *α*2 exhibit strong interaction, as indicated by a Fiberdock energy scoring of -42.36, whereas there were no predicted possible interactions with the intracytoplasmic tail *β*1 (**Figure 4C**).

**Table 3:** Correlation of mRNA expression levels of *ITGA2* and *ITGB1* with CC16 in bronchial epithelial cells in SARP.

Gene	Gene Annotation	Cross-Sectional Cohort (n = 155)		Longitudinal Cohort (n = 156)		Meta-Analysis*	
		rho	*P* value	rho	*P* value	rho	*P* value
**ITGA2**	VLA-2 subunit alpha 2	0.15	0.068	0.32	4.0x10^-5^	0.24	5.2x10^-6^
**ITGB1**	VLA-2 subunit beta 1	0.0056	0.94	0.087	0.28	0.046	0.21

*Meta-analysis of Spearman correlation coefficient (rho) was performed in the SARP cohorts (n=311) using metacor R package on the Olkin-Pratt fixed-effect meta-analytical model.

### VLA-2 inhibition results in decreased SPLUNC1 during Mp infection

**Figure 5:**
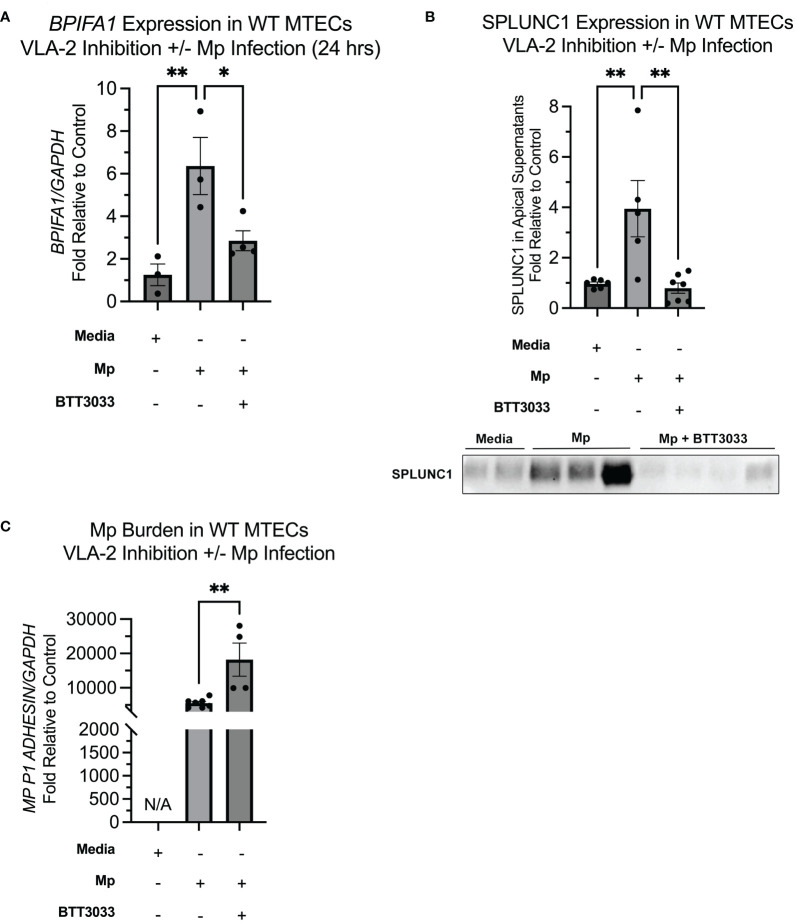
Inhibition of VLA-2 results in decreased SPLUNC1 expression and increased Mp burden. **(A)** *BPIFA1* gene expression was measured by RT-PCR in WT MTECs treated with media only (n=3), Mp (n=3), Mp+rCC16 (n=4), Mp+rCC16+BTT3033 (n=4), or Mp+BTT3033 (n=4). *GAPDH* was used as a housekeeping control. ^*^P<0.05, ^**^P<0.01 by One-Way ANOVA Tukey’s multiple comparison test. **(B)** Apical SPLUNC1 protein expression was measured in the same samples from panel *A*, as well as additional experimental samples to confirm reproducibility, by western blotting. A representative blot is shown. ^*^P<0.05, ^**^P<0.01 by One-Way ANOVA Tukey’s multiple comparison test. **(C)** Mp burden was measured in the same samples from panels *A* by RT-PCR. *GAPDH* was used as a housekeeping control. ^*^P<0.05, ^**^P<0.01 by One-Way ANOVA Tukey’s multiple comparison test. Data are presented as mean±SEM.

We next sought to determine the impact of CC16 and VLA-2 inhibition on SPLUNC1 expression in the context of Mp infection. Similar to what has been reported by Gally et al., we observed that upon infection with Mp, SPLUNC1 expression is significantly increased, compared to the media only control (**Figures 5A, B**) (40). Addition of BTT3033 during Mp infection, without exogenous rCC16, resulted in lower levels of SPLUNC1 expression, which we propose is likely due to inhibition of endogenous CC16 from signaling through VLA-2 (**Figures 5A, B**). The significantly decreased SPLUNC1 levels in the Mp+BTT3033 group correlated to significantly increased Mp burden, compared to control (**Figure 5C**), which is in line with previous publications that demonstrated SPLUNC1 activity against Mp infection (17).

### Mutant D67A rCC16 fails to increase SPLUNC1 expression in MTECs

**Figure 6:**
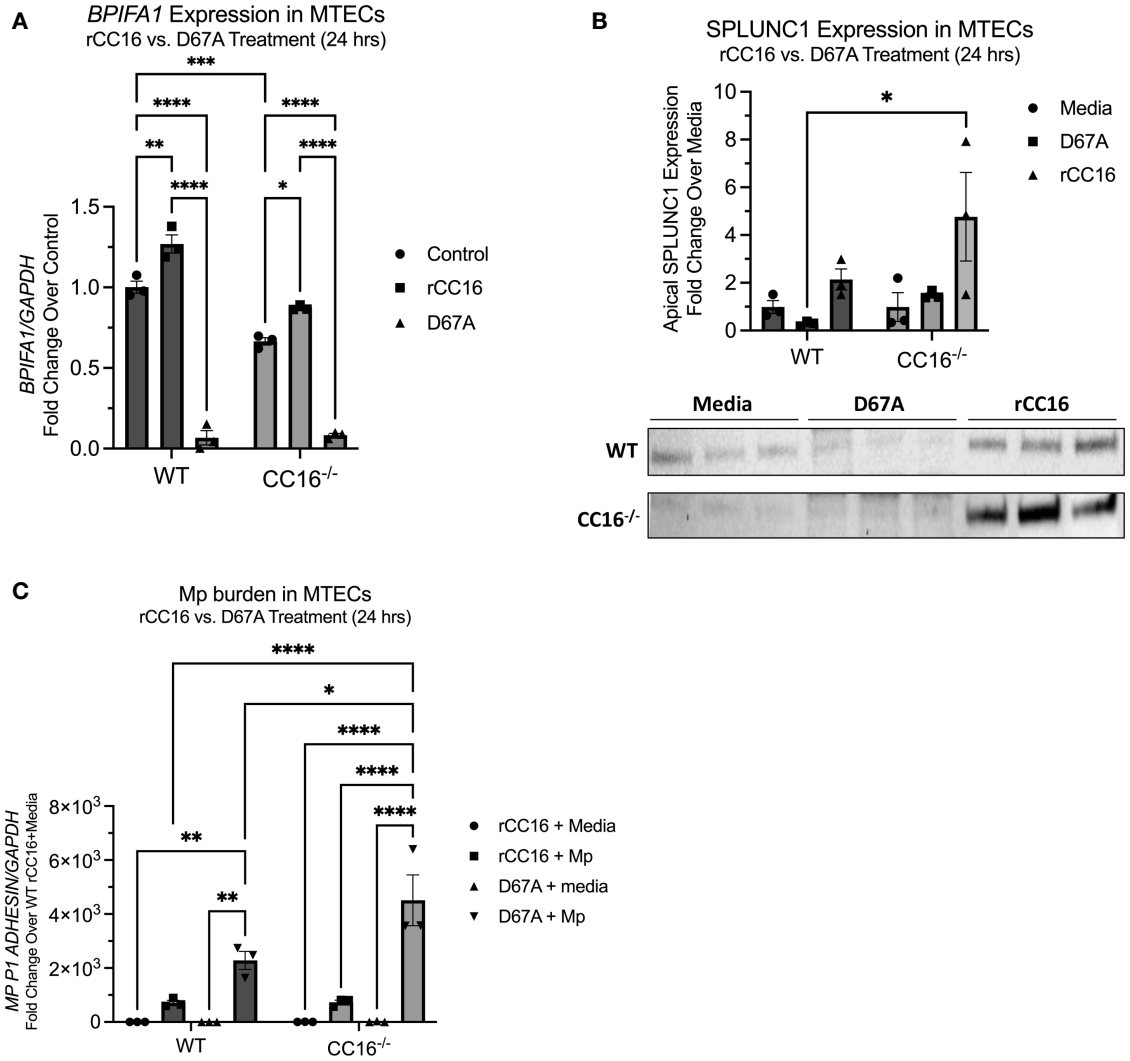
D67A rCC16 does not induce SPLUNC1 expression and results in increased Mp burden. **(A)** *Bpifa1* expression was measured by RT-PCR in WT and CC16^-/-^ MTECs that were treated with media, WT rCC16, and mutant D67A rCC16 for 24 hrs. Gapdh was used as a housekeeping control. ^*^P<0.05, ^**^P<0.01, ^***^P<0.001, ^****^P<0.0001 by Two-Way ANOVA Šídák’s multiple comparisons test. **(B)** SPLUNC1 expression was measured in the same samples as panel **(A)** by western blotting. ^*^P<0.05 by Two-Way ANOVA Šídák’s multiple comparisons test. **(C)** Mp burden was measured by RT-PCR in WT and CC16^-/-^ MTECs that were infected with Mp or treated with media during rCC16 and/or mutant D67A rCC16 treatment for 24 hrs. Gapdh was used as a housekeeping control. ^*^P<0.05, ^**^P<0.01, ^****^P<0.0001 by Two-Way ANOVA Šídák’s multiple comparisons test.

To determine if the LVD integrin binding site within CC16 is necessary for induction of SPLUNC1 expression via VLA-2 interactions, we treated WT and CC16^-/-^ MTECs with a mutant rCC16 that has a mutation in the leucine-valine-aspartic acid (LVD) motif as described previously (8). Our group has previously shown that this mutant rCC16 (D67A rCC16) is unable to bind to the VLA-4 integrin complex (8). Upon treatment with the mutant D67A rCC16, not only did the mutant fail to promote an increase in SPLUNC1, but we also actually observed significantly decreased SPLUNC1 expression by both WT and CC16^-/-^ MTECs (**Figures 6A, B**). In line with decreased SPLUNC1, we also observed significantly increased Mp burden from MTECs treated with D67A rCC16 (**Figure 6C**).

### rSPLUNC1 treatment decreases Mp burden in CC16^-/-^ mice

**Figure 7:**
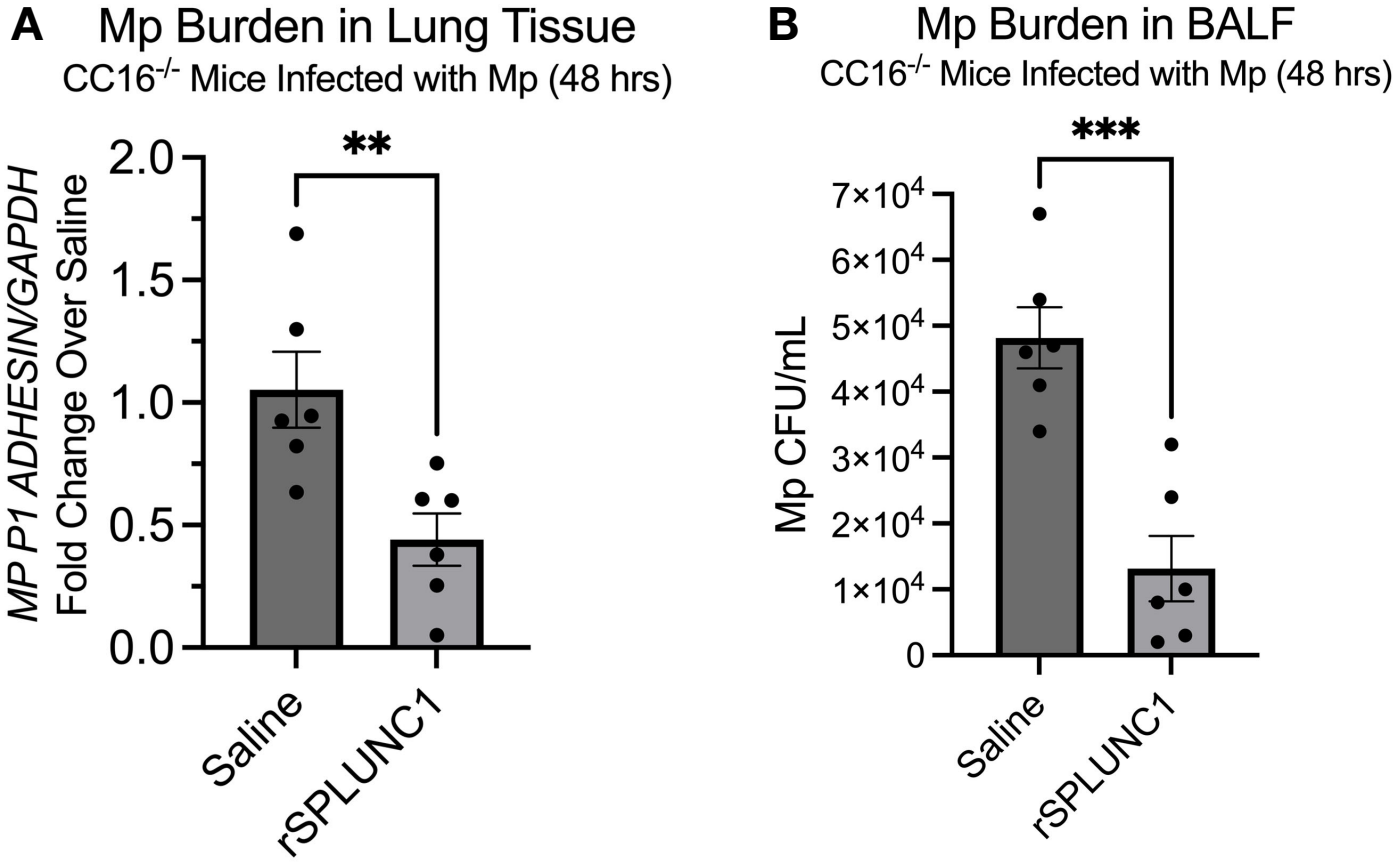
rSPLUNC1 treatment decreases Mp burden in CC16^-/-^ mice. **(A)** Mp burden was measured by RT-PCR in lung tissue from CC16^-/-^ infected with Mp, followed by rescue with rSPLUNC1 treatment. *GAPDH* was used as a housekeeping control. ^**^P<0.01 by Unpaired *t*-test. **(B)** Mp burden was measured in the BALF from these same mice in panel **(A)**; however, Mp burden was measured by plating samples on PPLO agar, followed by counting colonies, to determine Mp CFU/mL. ^***^P<0.001 by Unpaired *t*-test.

Since our data suggests that SPLUNC1 expression is dependent on the presence of CC16, we aimed to demonstrate that rSPLUNC1 treatment could decrease and “rescue” Mp burden in CC16^-/-^ mice. CC16^-/-^ mice were infected with Mp, followed by treatment with rSPLUNC. Mp burden was assessed in lung tissue and bronchoalveolar lavage fluid (BALF). Treatment with rSPLUNC1 resulted in significantly decreased Mp burden in both the lungs and BALF from the CC16^-/-^ mice (**Figures 7A, B**). These data suggest that rSPLUNC1 treatment may benefit individuals with decreased CC16 levels and recurrent Mp infections, such as asthma, COPD, and cystic fibrosis patients (10, 11, 17, 37, 41–47).

## Discussion

CC16 has been shown to protect from lung function deficits in the setting of infectious as well as non-infectious conditions, partly through its anti-inflammatory and antioxidant effects (7–10); however, the mechanisms by which CC16 exerts these effects, especially regarding the pulmonary epithelium, have not been fully elucidated. To better understand if and how CC16 regulates the expression of pulmonary epithelial-driven antimicrobial proteins, we utilized mass spectrometry and quantitative proteomics to identify apically secreted proteins from WT and CC16^-/-^ MTECs. Using this approach, we are the first, to our knowledge, to show that SPLUNC1 expression is induced by CC16 within the pulmonary epithelium, which we validated in human primary airway cells. Further, we show in MTECs and HNECs that CC16 signals through the VLA-2 integrin complex within the pulmonary epithelium to induce SPLUNC1 expression. The identification of VLA-2 as novel receptor for CC16 on the respiratory epithelia provides another layer of understanding for how CC16 provides protection in the airway by another direct integrin interaction, in addition to the interaction with VLA-4 on circulating leukocytes.

Since studies have previously defined that low serum and BALF CC16 levels associate with declines in lung function in patients (7, 10–12, 14), our work now sheds light on a novel mechanism by which low CC16 contributes to decreased expression of epithelial-driven antimicrobials, which likely results in more persistent respiratory infections and a decrease in lung function over time. Low SPLUNC1 has been shown to result in decreased ASL volume, altered mucociliary clearance, decreased lung function, and increased pathogen burden during infection (10–12, 14). Based on these previous publications in which SPLUNC1 activity has been defined, one can surmise that the clinical implications for individuals with low CC16 levels, such as asthma, COPD, and cystic fibrosis patients (10–12, 14), is that they may also have decreased pulmonary SPLUNC1 levels and therefore a myriad of SPLUNC1-deficiency related respiratory symptoms.

Previously, our group showed that CC16^-/-^ mice and MTECs had higher Mp burden after infection compared to their WT counterparts (8, 9). In support of our published findings, we identified 8 antimicrobial proteins from MTECs, 6 of which were corroborated by bronchial gene expression in the SARP cohorts, whose expression appears to be associated with the level of CC16, each of which may aid in the resolution of Mp infection, as well as additional respiratory pathogens. SPLUNC1 was the antimicrobial protein that was the most differentially regulated between WT MTECs and CC16^-/-^ MTECs; therefore, further studies were conducted to better understand how CC16 regulates the expression of SPLUNC1. SPLUNC1 has been shown to have direct antimicrobial activity against Mp (40); therefore, the decreased expression of this protein by CC16^-/-^ MTECs provides mechanistic insight into the observed increased Mp burden in these MTECs (9). Translationally, this suggests that therapeutics targeting at increasing or replacing CC16 could provide benefit to individuals that have decreased pulmonary SPLUNC1 levels, such as cystic fibrosis patients (47–49), asthmatics (44, 50–52), and chronic obstructive pulmonary disease (COPD) patients (19, 53, 54), as these individuals may have decreased clearance of Mp infections leading to increased pathogen burden and decreased lung function. Interestingly, and supportive of our findings, each of these diseased populations have been shown to have decreased CC16 levels as well (7, 10–12), although the mechanistic connection between CC16 and SPLUNC1 had not been made.

To determine if CC16 could enhance expression of SPLUNC1, we added rCC16 to WT and CC16^-/-^ MTECs, after which we measured SPLUNC1 expression at the gene and protein level. rCC16-treated WT and CC16^-/-^ MTECs had significantly increased SPLUNC1 gene and protein expression, compared to their control-treated counterparts, indicating that CC16 can activate SPLUNC1 expression within the pulmonary epithelium and that CC16^-/-^ MTECs can generate SPLUNC1 if given exogenous CC16. We further confirmed this *in vivo*, by performing a rescue experiment by intravenously treating CC16^-/-^ mice for 3 days with rCC16 and assessing SPLUNC1 levels in BALF. We observed significantly increased SPLUNC1 levels in the BALF from CC16^-/-^ mice treated with rCC16, compared to saline-treated CC16^-/-^ mice. Interestingly, since we treated these mice intravenously, these data suggest that systemic rCC16 can rescue SPLUNC1 levels within the lung, which provides a path forward to consider therapeutic development of a systemic delivery as opposed to lung only delivery. Our group has similarly observed that intravenously administered rCC16 decreased airway hyperresponsiveness and resistance during methacholine treatment of Mp-infected WT and CC16^-/-^ mice (8); therefore, further supporting the claim that circulating CC16 exerts protective effects within the lung.

Correlation analysis in human data cohorts showed that CC16 and SPLUNC1 are significantly and positively correlated. Two human cohorts – SARP1-2 and SARP3 – were used to correlate *CC16* and *Splunc1* mRNA levels to ascertain clinical relevance. Asthmatics within the cohorts had positive Rho values and p values ≤ 0.05, confirming there is a significant and positive correlation between *SCGB1A1* and *BPIFA1* mRNA levels.

Since we determined that CC16 was not directly activating the *BPIFA1* promoter, or signaling through TLR2 to activate SPLUNC1, we next decided to focus on CC16 signaling through integrins on the pulmonary epithelium to induce SPLUNC1 expression. Our group has previously shown that CC16 binds to VLA-4 on leukocytes, thereby preventing extravasation into the lung and decreasing exuberant airway inflammation during Mp infection (8). While VLA-4 levels were very low to non-detectable in airway respiratory cells, using single cell RNA-seq data, we determined that VLA-2 is highly expressed within the pulmonary epithelium; therefore, we thought that it was highly likely that VLA-2 might be a previously undiscovered binding partner for CC16. Studies have shown that VLA-2 is not only highly expressed on airway bronchial epithelial cells but is also apically expressed; therefore, possibly playing a role in cell signaling (55–57). Based on this, we inhibited VLA-2 to determine if CC16 is signaling through this integrin complex to induce SPLUNC1 expression. We observed that VLA-2 was essential for rCC16 to increase SPLUNC1 expression in WT MTECs and healthy HNECs. Additionally, we show that expression of the alpha-2 (gene name: *ITGA2*), but not beta-1 (gene name: *ITGB1*), is significantly (5.4x10^-6^) and positively correlated to *CC16* expression in human bronchial epithelial cells, which suggests a potential greater interaction between CC16 and the alpha subunit of VLA-2.

Using “ISLAND: *In silico* protein affinity predictor” (30) we were able to predict the binding affinity of CC16 and VLA-2. These results showed that the binding affinity between CC16 and VLA-2, particularly the *α*2 subunit, was favorable (ΔΔG = -10.861 J) and the dissociation constant (Kd) exhibited strong binding (1.08x10^-8^ M). Furthermore, using *in silico* protein 3D structure models, we confirmed by Fiberdock energy scoring that CC16 preferentially binds to the α2 subunit of the α2β1 integrin receptor complex. This finding is in line with the human data from the SARP cohort that indicated significant associations between BEC CC16 expression and the α2, but not the β1, subunit. To further support this interaction, we treated WT and CC16^-/-^ MTECs with WT and mutant (D67A) rCC16 (8), followed by assessment of SPLUNC1 expression and Mp burden. As previously described, D67A rCC16 has a mutation in the leucine-valine-aspartic acid (LVD) binding motif, and we have shown that this mutation limits binding between CC16 and VLA-4 on leukocytes (8). Not only does our data show that treatment of MTECs with D67A inhibits SPLUNC1 expression, but it seems to repress expression of SPLUNC1 below baseline levels, although future studies are needed to better understand mechanisms driving this observation. Along these lines, we observed that the MTECs treated with the mutant D67A rCC16 had significantly increased Mp burden, which corresponds to the decreased SPLUNC1 expression within these samples. Taken together these data suggest that the previously detailed integrin binding site is also relevant for CC16’s interaction with VLA-2 within the pulmonary epithelium.

We next wanted to study the impact of CC16 and VLA-2 inhibition on Mp burden. As previously described by Chu et al., we observed that Mp infection increases expression of SPLUNC1 (17). We also observed that VLA-2 inhibition during Mp infection results in significantly decreased SPLUNC1 expression and subsequently increased Mp burden. We have previously shown that Mp infection increases expression of CC16 (8); therefore, these data suggest that VLA-2 inhibition was able to block endogenous CC16 from signaling during Mp infection.

We then went on to assess if rSPLUNC1 treatment would decrease Mp burden in the absence of CC16. We infected CC16^-/-^ mice with Mp, followed by treatment with rSPLUNC1 for 48 hrs. We observed that rSPLUNC1 treatment significantly decreased Mp burden within the lungs and BALF of these mice; therefore, rSPLUNC1 may be a viable treatment for individuals with decreased CC16 levels, such as asthma (8, 9, 11, 58), COPD (7, 10, 13, 45), and cystic fibrosis (14) patients, who have significantly decreased CC16 levels.

Overall, our data, to the best of our knowledge, is the first to show that CC16 induces the expression of a major pulmonary epithelial-driven antimicrobial protein, SPLUNC1, by signaling through VLA-2. We provided evidence that CC16 and SPLUNC1 are significantly and positively correlated within the pulmonary epithelium of mice and humans. Future studies are needed to determine if CC16 induces the expression of the other identified antimicrobial proteins (shown in **Figure 1**, **Table 1**) through this same mechanism of action or if there are other novel receptors and mechanisms by which CC16 is signaling within the pulmonary epithelium to promote host defense against respiratory infections. Additionally, further studies are needed to determine if there are potential sex differences regulating CC16 and SPLUNC1 expression, although none were detected in our studies using both male and female mice. Clinically, these results suggest that individuals with low CC16 may have decreased SPLUNC1 and higher susceptibility to Mp infection and that administration of CC16 as a therapeutic may be important to activate the expression of SPLUNC1 to aid in the induction of antimicrobial responses and decrease pathogen burden. Since several chronic respiratory diseases have been associated with low CC16 levels, including asthma (42, 43, 59–61), COPD (1, 6, 10, 46, 62–65), and cystic fibrosis (14), those individuals may be at an increased risk for respiratory infections due to loss of SPLUNC1 as well as low CC16. Taken together, our findings reinforce the potential of CC16 augmentation as a novel therapeutic approach to enhance resilience to respiratory infections among a variety of airway diseases.

## Data availability statement

The mass spectrometry proteomics data have been deposited to the ProteomeXchange Consortium via the PRIDE partner repository with the dataset identifier PXD046830 and 10.6019/PXD046830. Additionally, publicly available datasets were analyzed in this study. For the single-cell sequencing data, this dataset can be found here: GitHub [https://github.com/seiboldlab/SingleCell_smoking].

## Ethics statement

The studies involving humans were approved by Institutional Review Board at the University of Arizona. The studies were conducted in accordance with the local legislation and institutional requirements. The participants provided their written informed consent to participate in this study. The animal study was approved by Institutional Animal Care and Use Committee at the University of Arizona. The study was conducted in accordance with the local legislation and institutional requirements.

## Author contributions

NI: Conceptualization, Data curation, Formal Analysis, Funding acquisition, Investigation, Methodology, Software, Validation, Visualization, Writing – original draft, Writing – review & editing. HW: Data curation, Formal Analysis, Writing – review & editing. NL: Data curation, Formal Analysis, Writing – review & editing. MJ: Formal Analysis, Resources, Writing – review & editing. JR-Q: Data curation, Methodology, Visualization, Writing – review & editing. FP: Data curation, Formal Analysis, Writing – review & editing. SG: Conceptualization, Funding acquisition, Investigation, Resources, Supervision, Writing – review & editing. XL: Data curation, Formal Analysis, Software, Writing – review & editing, Investigation. DC: Data curation, Formal Analysis, Writing – review & editing, Validation. PL: Data curation, Formal Analysis, Methodology, Resources, Software, Supervision, Writing – review & editing. JL: Conceptualization, Formal Analysis, Funding acquisition, Investigation, Methodology, Project administration, Resources, Supervision, Validation, Visualization, Writing – original draft, Writing – review & editing.

## Supplementary material

Supplementary Figure 1 Intracellular SPLUNC1 is not impacted by CC16 deficiency. SPLUNC1 protein expression was confirmed for control-treated WT and CC16^-/-^ MTECs lysates (n=3), and CC16^-/-^ mice (n=4) by western blotting. The same amount of protein (10 *μ*g) was loaded for each sample. SPLUNC1 protein levels are normalized to GAPDH. Data are presented as mean±SEM.

Supplementary Figure 2 Scatter plot of *SCGB1A1* and *BPIFA1* normalized expression within mucus secretory cells. Each point represents an individual cell. Pearson correlation coefficient (p-value = 9.7 x10^-31^) displayed above graph. The blue line is a regression line fit to the expression values of these two genes.

Supplementary Figure 3 Representative major epithelial cell populations in WT and CC16^-/-^ mouse lungs by flow cytometry. Club (CYP2F2), ciliated (TUBA1A), goblet (MUC5AC), and basal (KRT5) cell percentages were measured in WT *(A)* and CC16^-/-^ *(B)* mouse lungs by flow cytometry. Alexa Flour 647 was used as the secondary antibody for detection of club, ciliated, and goblet cells. Alexa Fluor 488 was used as the secondary antibody for detection of basal cells. Gray histograms represent unstained cells; black histograms represent isotype controls; and blue histograms represent experimental antibody.

Supplementary Figure 4 Representative major epithelial cell populations in WT and CC16^-/-^ MTECs by flow cytometry. Club (CYP2F2), ciliated (TUBA1A), goblet (MUC5AC), and basal (KRT5) cell percentages were measured in WT *(A)* and CC16^-/-^ *(B)* MTECs by flow cytometry. Alexa Flour 647 was used as the secondary antibody for detection of club, ciliated, and goblet cells. Alexa Fluor 488 was used as the secondary antibody for detection of basal cells. Gray histograms represent unstained cells; black histograms represent isotype controls; and blue histograms represent experimental antibody.

Supplementary Figure 5 CC16 does not directly activate the SPLUNC1 promoter. NCI-H292 were transfected with a SPLUNC1 promoter reporter construct containing a novel luminescent reporter gene (RenSP luciferase). After transfection (24 hrs), media or rCC16 (*A*), apically secreted proteins from WT and CC16^-/-^ MTECs (*B*), and WT and CC16^-/-^ MTEC lysates (*C*) were added to the transfected NCI-H292 cells (24 hrs), after which promoter activity was measured by luminescence. An actin beta (ACTB) promoter vector was used as a positive control; a random genomic fragment promoter vector was used as a negative control; and the SPLUNC1 promoter vector was used as the experimental condition. ^****^*P<0.0001* by Two-Way ANOVA Šidák’s multiple comparison test. Data are presented as mean±SEM.

Supplementary Figure 6 CC16 activation of SPLUNC1 expression is independent of TLR2. *(A)* WT MTECs (n=4; except α-TLR2+rCC16 n=3) were treated with either a TLR2 blocking antibody (α-TLR2) or an isotype control antibody (IgG1) (10 *μ*g/mL) for 30 min, followed by treatment with media only, rCC16 (25 *μ*g/mL), or Pam3CSK4 (1 *μ*g/mL) for 24 hrs. Following the treatments, *Splunc1* gene expression was measured by RT-PCR with *Gapdh* as a housekeeping control. *^*^P<0.05* by One-Way ANOVA Tukey’s multiple comparison test. *(B)* SPLUNC1 apical protein expression was assessed for media-, rCC16-, and Pam3CSK4-treated WT MTECs following incubation with an *α*-TLR2 antibody or IgG1 isotype antibody (10 *μ*g/mL) by western blotting (n=3 per group). The same volume of protein (20*μ*l) was loaded for each sample. ^**^*P<0.01*, ^****^*P<0.0001* by One-Way ANOVA Tukey’s multiple comparison test.
