# Supplementary material for: CC16 drives VLA-2-dependent SPLUNC1 expression
Source: Front Immunol. 2023 Nov 20;14:1277582. doi: 10.3389/fimmu.2023.1277582 (PMC10694244; doi:10.3389/fimmu.2023.1277582)
Supplement: Supplementary file 1 [file DataSheet_1.docx]

**SUPPLEMENTAL METHODS**

**Experimental Mice**

WT and CC16^-/-^ male mice on a C57BL/6J background were obtained at ~6-8 weeks of age at the time of rCC16 treatment, or males and females >12 weeks of age for MTECs as more cells are recovered for growth on Transwells from aged mice. All mice were born and raised in the same room in the University of Arizona Health Sciences animal facility and were tested to be specific-pathogen free according to standard protocols using sentinel mice from the same room.

**NCI-H292 Cell Culturing**

NCI-H292 cells (human pulmonary mucoepidermoid carcinoma cells) (ATCC; Manassas, VA) were cultured in T75 flasks using ATCC-formulated RPMI-1640 medium (ATCC 30-2001) supplemented with 10% FBS, 100 U/mL penicillin and 100 μg/mL streptomycin at 37^o^C, 5% CO_2_. Cell culture medium was replaced every 48 hrs. Once 90% confluency was reached, the cells were removed from by flask by incubation (37^o^C, 5% CO_2_) with 0.25% trypsin-EDTA for 10 min. Cells were passaged (1:8 dilution) into fresh RPMI-1640 medium supplemented with 10% FBS.

**Human Nasal Epithelial Cell Isolation and Culturing**

Human nasal epithelial cell (HNEC) brushings were obtained from healthy donors under University of Arizona IRB-approved protocols (PI: Ledford). After collection, HNECs were submerged in sputolysin (MilliporeSigma; Burlington, MA) diluted in RPMI supplemented with an antibiotic/antimycotic (Gibco; Waltham, MA) for 30 min (37^o^C, 5% CO_2_) followed by centrifugation (350 RCF, 5 min, 4^o^C). The HNECs were washed three times using 10% FBS/RPMI followed by centrifugation (350 RCF, 5 min, 4^o^C). HNECs were seeded in collagen coated T25 flasks at a density of 5x10^5^ cells per 25 cm^2^ using PneumaCult Ex-Plus media (StemCell Technologies; Vancouver, CA). HNECs were removed from the T25 flasks by incubation (37^o^C, 5% CO_2_) with ACF enzymatic dissociation solution for 7-8 min, followed by addition of ACF enzyme inhibition solution to stop the reaction. Cells were plated onto Costar Transwell membranes (12 mm, 0.4 μm membrane pores) at a density of 89,600 cells per membrane in PneumaCult Basal ALI or Basal media (StemCell Technologies; Vancouver, VA) as needed. When the cells reached 80% confluency (~5 days), the culture medium was replaced daily, only on the basolateral side, to establish an ALI. After the first day in an ALI, the medium was replaced every 48 hrs. Once full confluency was obtained, the cells were maintained at an ALI for 14 days.

**Mouse Tracheal Epithelial Cell (MTEC) Isolation**

Mice were humanely euthanized, and tracheas were removed by dissection and placed in Ham’s F-12 medium on ice. Any excess connective tissue, muscle, vasculature, and nerves were removed from the exterior of the tracheas, and a longitudinal incision was made to expose the mucosal lining. The tracheas were placed in 10 mL DMEM F-12 medium (Gibco; Waltham, MA) with 0.1% protease solution (Sigma-Aldrich; St. Louis, MO) and incubated for 45 min at 37^o^C. The protease activity was stopped by adding 3 mL FBS (HyClone; Logan, UT). The tracheas were then transferred to a petri dish containing DMEM F-12 medium, and the mucosal lining of each trachea was scraped using a 10 μL pipette tip. The cells were collected, transferred to a 15-mL conical tube, and centrifuged (900 rpm, 5 min, 4^o^C). The medium supernatant was removed, and the cell pellet was resuspended in 5 mL Versene (Life Technologies; Waltham, MA) for 15 min at 37^o^C. Lastly, MTECs were centrifuged (900 rpm, 5 min, 4^o^C), resuspended in Keratinocyte Serum Free Media (KSFM) (Gibco; Waltham, MA), and seeded for growth in T75 tissue culture-treated flasks (Corning; Corning, NY).

**MTEC Culture Media and Supplements**

KSFM was used for MTEC expansion in T75 flasks prior to seeding on transwell membranes for air-liquid interface (ALI) culturing. KSFM for MTEC expansion was supplemented with 100 U/mL penicillin and 100 μg/mL streptomycin (Gibco; Waltham, MA), 0.025 μg/mL murine epidermal growth factor (EGF) (Sigma-Aldrich; St. Louis, MO), 0.03 mg/mL bovine pituitary extract (BPE) (Lonza; Basel, Switzerland), 1 μM isoproterenol (Sigma-Aldrich; St. Louis, MO), 5$\mu$M DAPT (Sigma-Aldrich; St. Louis, MO), and 10$\mu$M Y-27632 (BD Biosciences; Franklin Lakes, NJ).

DMEM F-12 growth media was used for the trachea harvest and growth of ALI MTEC cultures. DMEM F-12 growth media was supplemented with 100 U/mL penicillin and 100 μg/mL streptomycin, 0.03% (w/v) sodium bicarbonate (Gibco; Waltham, MA), 1.5 mM L-glutamine (Gibco; Waltham, MA), 5% FBS, 1x ITS-G (Gibco; Waltham, MA), 0.1 μg/mL cholera toxin (Sigma-Aldrich; St. Louis, MO), 0.025 μg/mL murine EGF, 0.03 mg/mL BPE, 10$\mu$M Y-27632, and 0.05$\mu$M retinoic acid (Sigma-Aldrich; St. Louis, MO).

DMEM F-12 differentiation media was used for the differentiation of ALI MTEC cultures. DMEM F-12 differentiation media was supplemented with 100 U/mL penicillin and 100 μg/mL streptomycin, 0.03% (w/v) sodium bicarbonate, 1.5 mM L-glutamine, 0.1% (w/v) bovine serum albumin (BSA) (Gibco; Waltham, MA), 1x ITS-G, 0.1 μg/mL cholera toxin, 0.025 μg/mL murine EGF, 0.03 mg/mL BPE, 0.05$\mu$M retinoic acid.

***In vitro* Culturing of MTECs**

*In vitro* MTEC expansion and culturing was based on the protocol published by Eenjes et al., which allowed for increased MTEC counts, while using less mice; thereby reducing biological variation [26]. MTECs (375,000 cells) were seeded in T75 tissue culture-treated flasks and grown at 37^o^C, 5% CO_2_ until 90% confluency was reached. Once confluent, MTECs were removed from the flasks by incubation (37^o^C, 5% CO_2_) with 0.25% tryspin-EDTA (Gibco; Waltham, MA) for 10 min. After removal from the flasks, MTECs were centrifuged (900 rpm, 5 min, 4^o^C) and resuspended in DMEM-F12 growth media for seeding on Transwell membranes. Costar Transwell (12 mm, 0.4 μm membrane pores) 12-well plates were used to culture the MTECs. The polyester membrane was coated with 300 μg/mL rat rail collagen in 0.02 N glacial acetic acid at room temperature for 1 hr. The membranes were washed with PBS and conditioned with DMEM F-12 growth media for 1 hr at 37^o^C. The media was then removed from the apical and basolateral sides, and 1 mL DMEM F-12 growth media was added only to the basolateral side of each well. The MTECs that were expanded in the T75 flasks were resuspended in DMEM F-12 growth media and 89,600 cells were plated on each Transwell membrane. For the MTECs to seed onto the Transwell membranes, the MTECs were incubated (37^o^C, 5% CO_2_) for 48 hrs without changing the media. After the initial seeding period, the DMEM F-12 growth media on the apical and basal sides of the membrane was replaced every other day. When the cells reached 80% confluency (~1 week), the culture medium was replaced daily, only on the basolateral side, to establish an ALI. After the first day in an ALI, the medium was changed to DMEM F-12 differentiation media and replaced every 48 hrs. Once full confluence was obtained, the cells were maintained at ALI for 14 days.

**In-Solution Tryptic Digestion**

In-solution tryptic digestion of the apically secreted proteins from WT and CC16^-/-^ MTECs was performed as described [27]. 100 μg of protein was subjected to acetone precipitation by adding six times the sample volume of pre-chilled 100 % acetone and incubated one hour at -20° C. The precipitates were centrifuged at 16,000 x g for 10 minutes at 4°C and the acetone was removed. 400 μL of pre-chilled 90% acetone was added to the protein pellet, briefly vortexed and centrifuged at 16,000 x g for 5 minutes at 4°C. The remaining acetone was removed, the protein pellets were air dried for 3 minutes, resuspended in 100 μL of 50 mM NH4HCO3 and sonicated for 5 minutes. The samples were supplemented with dithiothreitol (DTT) at a final concentration of 5 mM and incubated at 70° C for 30 minutes. Samples were cooled to room temperature for 10 minutes and incubated with 15 mM acrylamide for 30 minutes at room temperature while protected from light. The reaction was quenched with DTT with a final concentration of 5 mM and incubated in the dark for 15 minutes. One μg of Lys-C was added to each sample and incubated at 37° C for 2-3 hours while shaking at 300 rpm followed by the addition of 50 μL of 50mM ammonium bicarbonate and 2 $\mu$g of trypsin and incubation overnight at 37° C while shaking at 300 rpm. 14.7 µL of 40% FA/1% HFBA was added to each sample and incubated for 10 minutes (final concentration is 4% FA/0.1% HFBA) to stop trypsin digestion. The samples were desalted with Pierce Peptide Desalting Spin Columns per the manufacturer’s protocol (ThermoFisher Scientific, cat no. 89852) and the peptides were dried by vacuum centrifugation. The dried peptides were resuspended in 20 μL of 0.1% FA (v/v) and the peptide concentration was determined with the Pierce Quantitative Colorimetric Peptide Assay Kit per the manufacturer’s protocol (ThermoFisher Scientific, cat no. 23275). 350 ng of the final sample was analyzed by mass spectrometry.

**Mass Spectrometry and Data Search**

HPLC-ESI-MS/MS was performed as previously described [28] in positive ion mode on a Thermo Scientific Orbitrap Fusion Lumos tribrid mass spectrometer fitted with an EASY-Spray Source (Thermo Scientific, San Jose, CA). NanoLC was performed using a Thermo Scientific UltiMate 3000 RSLCnano System with an EASY Spray C18 LC column (Thermo Scientific, 50cm x 75 μm inner diameter, packed with PepMap RSLC C18 material, 2 µm, cat. #ES803); loading phase for 15 min at 0.300μL/min; mobile phase, linear gradient of 1–34% Buffer B in 119 min at 0.220 μL /min, followed by a step to 95% Buffer B over 4 min at 0.220 μL /min, hold 5 min at 0.250 μL/min, and then a step to 1% Buffer B over 5 min at 0.250 μL /min and a final hold for 10 min (total run 159 min); Buffer A = 0.1% FA/H2O; Buffer B = 0.1% FA in 80% ACN. All solvents were liquid chromatography mass spectrometry grade. Spectra were acquired using XCalibur, version 2.3 (ThermoFisher Scientific).

**Label-free Quantitative Proteomics**

Progenesis QI for proteomics software (version 2.4, Nonlinear Dynamics Ltd., Newcastle upon Tyne, UK) was used to perform ion-intensity based label-free quantification as previously described [29]. In an automated format, .raw files were imported and converted into two-dimensional maps (y-axis = time, x-axis =m/z) followed by selection of a reference run for alignment purposes. An aggregate data set containing all peak information from all samples was created from the aligned runs, which was then further narrowed down by selecting only +2, +3, and +4 charged ions for further analysis. The samples were then grouped in wild type versus knockdown. Peak lists of fragment ion spectra were exported in Mascot generic file (.mgf) format and searched against the Swissprot Mus musculus database (17097 entries) using Mascot (Matrix Science, London, UK; version 2.6). The search variables that were used were: 10 ppm mass tolerance for precursor ion masses and 0.5 Da for product ion masses; digestion with trypsin; a maximum of two missed tryptic cleavages; variable modifications of oxidation of methionine and phosphorylation of serine, threonine, and tyrosine; 13C=1. The resulting Mascot .xml file was then imported into Progenesis, allowing for peptide/protein assignment, while peptides with a Mascot Ion Score of <25 were not considered for further analysis. Precursor ion-abundance values for peptide ions were normalized to all proteins. For quantification, proteins must have possessed at least one or more unique identifying peptide.

**Determination of SPLUNC1 and Integrin Protein Levels by Western Blotting**

The right lung from mouse samples were homogenized in RIPA (radioimmunoprecipitation assay) buffer (Teknova; Hollister, CA) with protease inhibitors (Roche; Basel, Switzerland). After homogenization, the lungs were centrifuged at 12,000 rpm for 10 min at 4^o^C, and the supernatant was collected. Protein concentrations from lung lysates were quantified using a Pierce BCA Protein Assay Kit (Thermo Fisher Scientific; Waltham, MA). Equal amounts of lysate were loaded onto Mini-Protean TGX precast gels (Bio-Rad Laboratories; Hercules, CA). To determine SPLUNC1 protein levels in bronchoalveolar lavage fluid (BALF) from mice and apical secretions from MTECs, equal volumes of BALF and apical secretions were loaded into Mini-Protean TGX precast gels, respectively. To determine integrin protein levels in MTECs, a standard concentration (10 $\mu$g/mL) was loaded into each well of a Mini-Protean TGX precast gel. Antibodies for. All primary antibodies were diluted 1:1000 in 5% (w/v) nonfat dry milk-1X TBST and required an anti-rabbit secondary antibody (Cell Signaling; Danvers, MA). The secondary antibody was diluted 1:2000 in 5% (w/v) nonfat dry milk-1X TBST. A ChemiDoc imaging system and Image Lab software (Thermo Fisher Scientific; Waltham, MA) were used to image and quantify the densitometry of each western blot.

**Human Tracheal Epithelium Single-cell RNA Sequencing Data Analysis**

Single-cell RNA sequencing data of epithelial cells isolated from six healthy adult tracheal donors described in Goldfarbmuren et al. were used to assess the expression level and cell-type specificity of genes of interest [30]. An RData file with the data stored in a Seurat object with processed counts, cell IDs, and metadata was downloaded from Gene Expression Omnibus (GEO) - accession number GSE134174 [31]. The Seurat object was subset on healthy adult tracheal donor cells by retaining “never” from the metadata table column labeled “smokers” and adults (>18 years) from the “age” column. Data were normalized using the ‘NormalizeData’ function from Seurat. The top 2,000 variable genes were identified with the ‘FindVariableFeatures’ function, implementing a variability stabilizing transformation by using the selection.method = "vst" parameter. Data were subsequently scaled using the ‘ScaleData’ function. Dot plots were generated with the ‘DotPlot’ function with the argument scale.by = “size” and group.by = "cluster_ident" to group cells using the cell type annotations provided by the authors of the original study. We utilized the R package ‘Libra’ [PMID: 34584091] to define differentially expressed genes with the function ‘run_de’ and the metadata columns "cluster_ident" defining the cell types and “donor” as replicates to perform the likelihood-ratio test implemented in edgeR. Pearson correlation was found by first subsetting the Seurat object on “Mucus.secretory” cells and then running the function ‘cor.test’ between the rows *SCGB1A1* and *BPIFA1* in the processed gene by cell matrix.

**Flow Cytometry on WT and CC16^-/-^ Mouse Lungs and MTECs**

Mouse Lung Digestion and Staining for Flow Cytometry: Mice were humanely euthanized, and the lungs were perfused by injecting 20 mL HBSS into the right ventricle to remove red blood cells. The right and left lung lobes were collected, placed into a 60 mm non-tissue culture treated dish, and chopped with a razor blade until the lung pieces were 1-2 mm. HBSS+/+ (with Ca^2+^ and Mg^2+^) and DNaseI (0.2 mg/mL) were added to the lung pieces in each tissue culture dish, followed by addition of collagenase A (1 mg/mL) to each dish. The lungs were incubated for 1 hr at 37^o^C. At the end of the incubation, EDTA Stop Solution (222 mM EDTA in FBS + 56% FBS) was added to each sample to stop the digestion. The lungs were filtered through a 40 $\mu$M cell strainer and centrifuged for 10 min at 1,200 rpm (4^o^C). The supernatant was discarded, and the cell pellet was resuspended in 1x red blood cell lysis buffer for 5 min on ice, followed by centrifugation for 5 min at 1,200 rpm, and resuspension of the cell pellet in FACS buffer (1x PBS + 5% FBS). A cell count was performed to ensure each sample had 1x10^6^ cells/mL. The cells were fixed with 10% formalin for 15 min on ice, followed by washing the cells with FACS buffer (2 mL) one time (2,200 rpm, 5 min, 4^o^C). The cells were permeabilized with permeabilization buffer (1x PBS + 0.2% Triton X-100 + 5% FBS) for 10 min at room temperature, followed by washing the cells as previously described. The cells were blocked with blocking buffer (1x PBS + 5% goat serum + 1% BSA + 0.2% Triton X-100) for 30 min on ice, followed by addition of the primary antibodies against club (CYP2F2) (Santa Cruz Biotechnology; Dallas, TX), ciliated (TUBA1A) (Cell Signaling; Danvers, MA), goblet (MUC5AC) (Invitrogen; Waltham, MA), and basal (KRT5) (VWR; Radnor, PA) cells for 1-2 hrs at 4^o^C. After primary antibody incubation, the cells were washed as previously described, followed by addition of the secondary antibodies (AF488 and AF647 – 1:500 dilution) in blocking buffer for 1 hr, in the dark, at room temperature. The cells were washed as described previously, resuspended in 500 $\mu$l FACS buffer, and analyzed using an Attune NxT flow cytometer (Beckman Coulter).

MTEC Preparation and Staining for Flow Cytometry: Differentiated MTECs on transwell membranes were apically washed one time with 1x PBS to remove any traces of serum and mucus produced by the cells. Accumax was added to the apical and basolateral compartments of the MTECs and incubated (37^o^C) for 1 hr. After the Accumax incubation, the cells were removed from the transwell membranes, centrifuged at 1,200 rpm for 10 min (4^o^C), and resuspended in FACS buffer. A cell count was performed to ensure each sample had 0.5x10^6^ cells/mL - 1x10^6^ cells/mL. The MTECs were stained for flow cytometry following the same protocol as described for the mouse lungs.

**TLR2 Blocking Assay in MTECs**

Differentiated WT MTECs were apically washed once time with 1x PBS to remove any traces of serum and mucus produced by the cells. An $\alpha$-TLR2 antibody (Invivogen; San Diego, CA) or IgG1 isotype control (10 $\mu$g/mL) (R&D Systems; Minneapolis, MN) was apically added to the cells, followed by incubation for 30 min (37^o^C, 5% CO_2_). After incubation, media only (control), rCC16 (25 $\mu$g/mL), or Pam3CSK4 (1 $\mu$g/mL) (Invivogen; San Diego, CA) was apically added to the cells for 24 hrs (37^o^C, 5% CO_2_). After 24 hrs, the apically and basally secreted proteins, as well as RNA was collected from the cells.

***Splunc1* Luciferase Reporter Assay**

The LightSwitch Luciferase Assay System (SwitchGear Genomics; Carlsbad, CA) was used to transfect a *Splunc1* promoter reporter construct into NCI-H292 cells and assess if CC16 activates *Splunc1* gene expression directly and if CC16^-/-^ MTECs have altered activation of the *Splunc1* promoter. In short, NCI-H292 cells (5x10^3^ cells) were seeded in white 96-well tissue culture plates and incubated overnight (37^o^C, 5% CO_2_). The FuGENE HD Transfection Reagent (SwitchGear Genomics; Carlsbad, CA), Opti-MEM serum free media (ThermoFisher Scientific; Waltham, MA), and *Splunc1* GoClone plasmid DNA construct (30 ng/$\mu$L) (SwitchGear Genomics; Carlsbad, CA) were combined to achieve a 3:1 FuGENE HD:plasmid DNA ratio and allowed to sit at room temperature for 30 min. Afterwards, 5 $\mu$L of the transfection mix (FuGENE HD, plasmid DNA, OptiMEM combination) was added onto the seeded cells and incubated (37^o^C, 5% CO_2_) for 24 hrs. After 24 hrs, media (control), rCC16 (25 $\mu$g/mL), WT MTEC lysate (200 $\mu$g/mL), CC16^-/-^ MTEC lysate (200 $\mu$g/mL), WT apical supernatant (200 $\mu$g/mL), or CC16^-/-^ apical supernatant (200 $\mu$g/mL) was added to the seeded cells and incubated (37^o^C, 5% CO_2_) for 24 hrs. After 24 hrs, the LightSwitch Assay Reagent (SwitchGear Genomics; Carlsbad, CA) was prepared and added to the seeded cells (100 $\mu$L/well). The 96-well plate was covered to protect it from light and incubated at room temperature for 30 min. After incubation, each well was read for 2 seconds in a Biotek Synergy HTX plate luminometer (Agilent; Santa Clara, CA).
