# Supplementary figures and images for: CC16 drives VLA-2-dependent SPLUNC1 expression
Source: Front Immunol. 2023 Nov 20;14:1277582. doi: 10.3389/fimmu.2023.1277582 (PMC10694244; doi:10.3389/fimmu.2023.1277582)

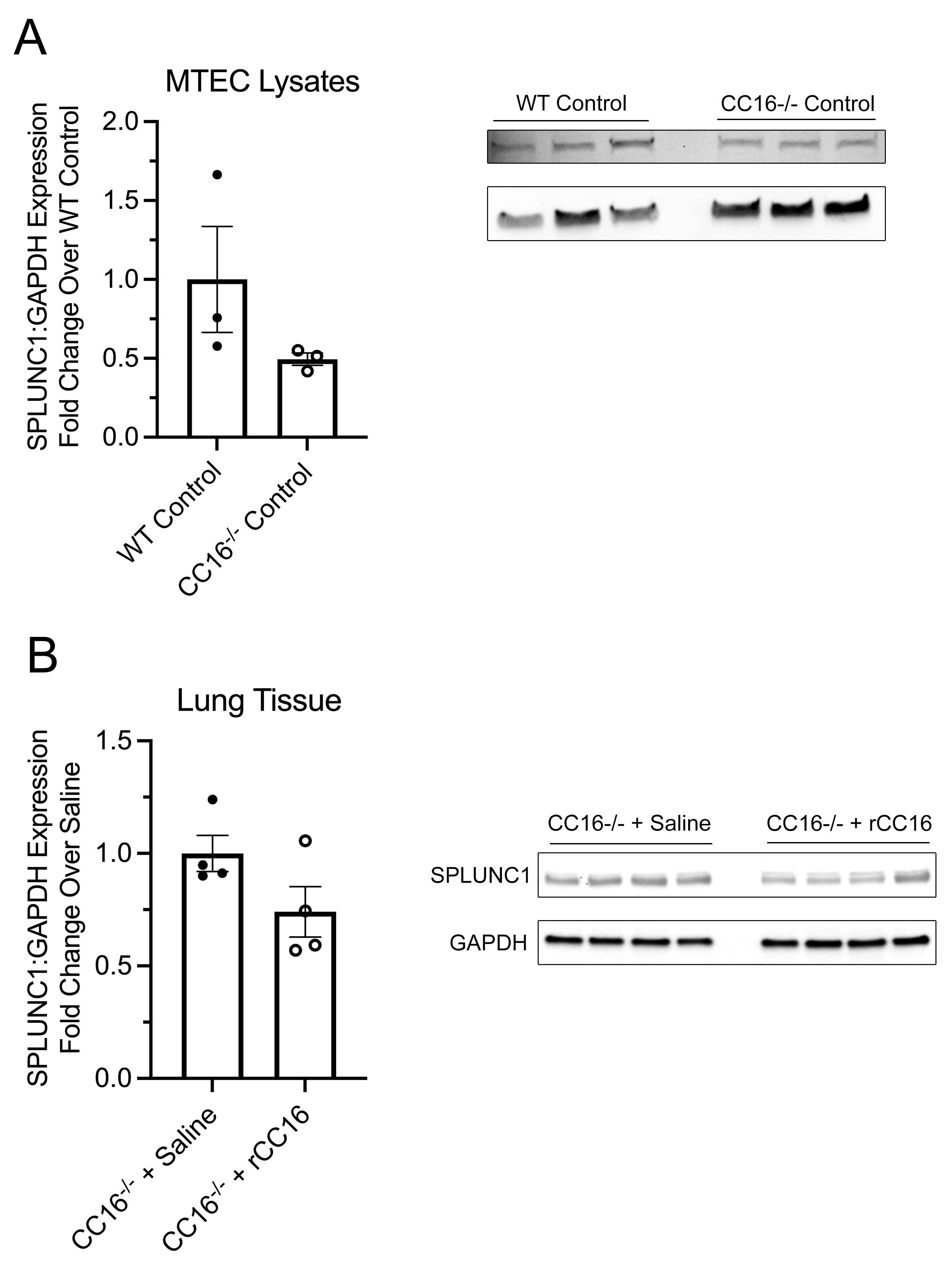

Supplement: Supplementary Figure 1 — Intracellular SPLUNC1 is not impacted by CC16 deficiency. SPLUNC1 protein expression was confirmed for control-treated WT and CC16-/- MTECs lysates (n=3), and CC16-/- mice (n=4) by western blotting. The same amount of protein (10 μg) was loaded for each sample. SPLUNC1 protein levels are normalized to GAPDH. Data are presented as mean±SEM. [file Image_1.tif]

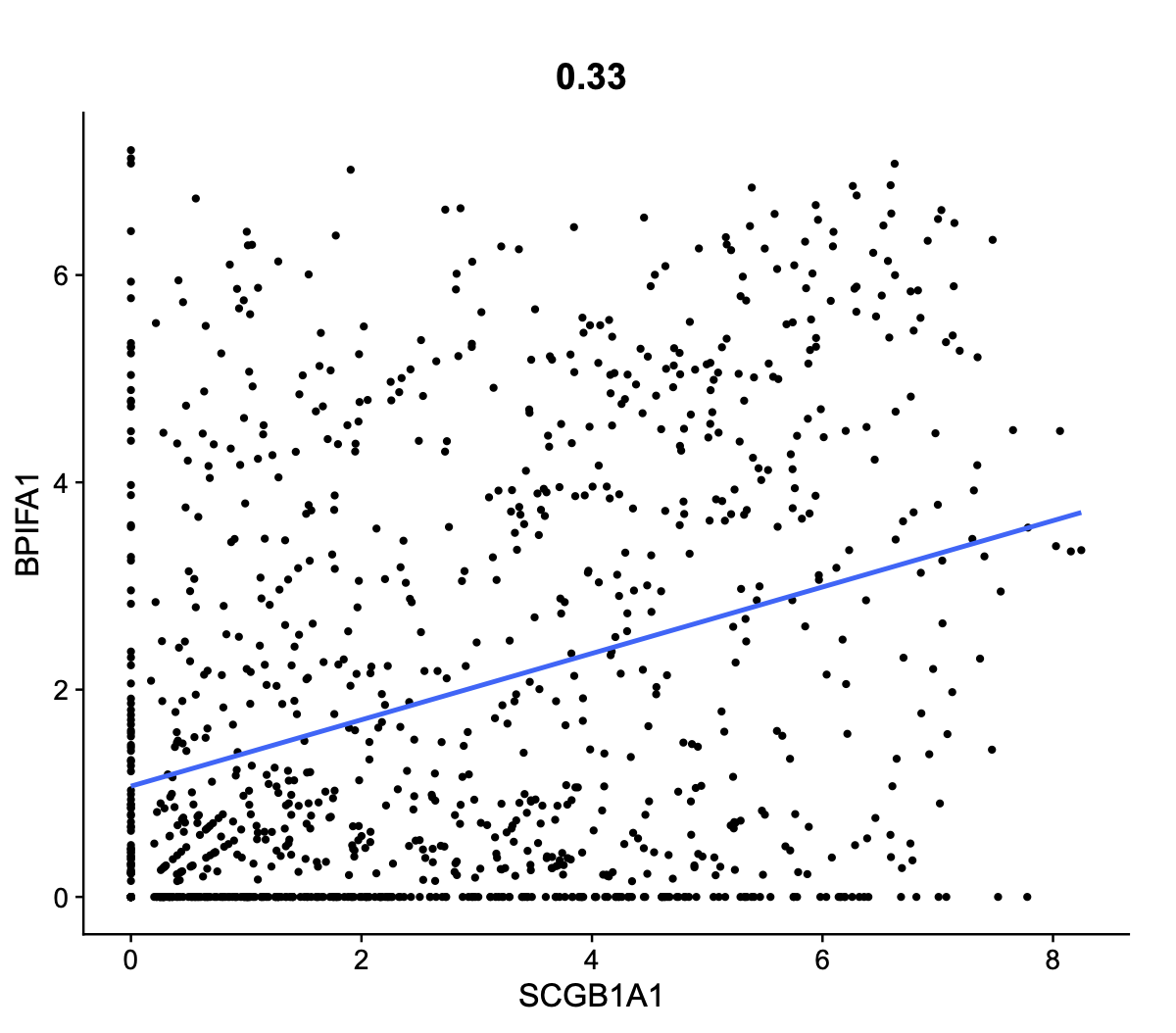

Supplement: Supplementary Figure 2 — Scatter plot of SCGB1A1 and BPIFA1 normalized expression within mucus secretory cells. Each point represents an individual cell. Pearson correlation coefficient (p-value = 9.7 x10^-31^) displayed above graph. The blue line is a regression line fit to the expression values of these two genes. [file Image_2.tiff]

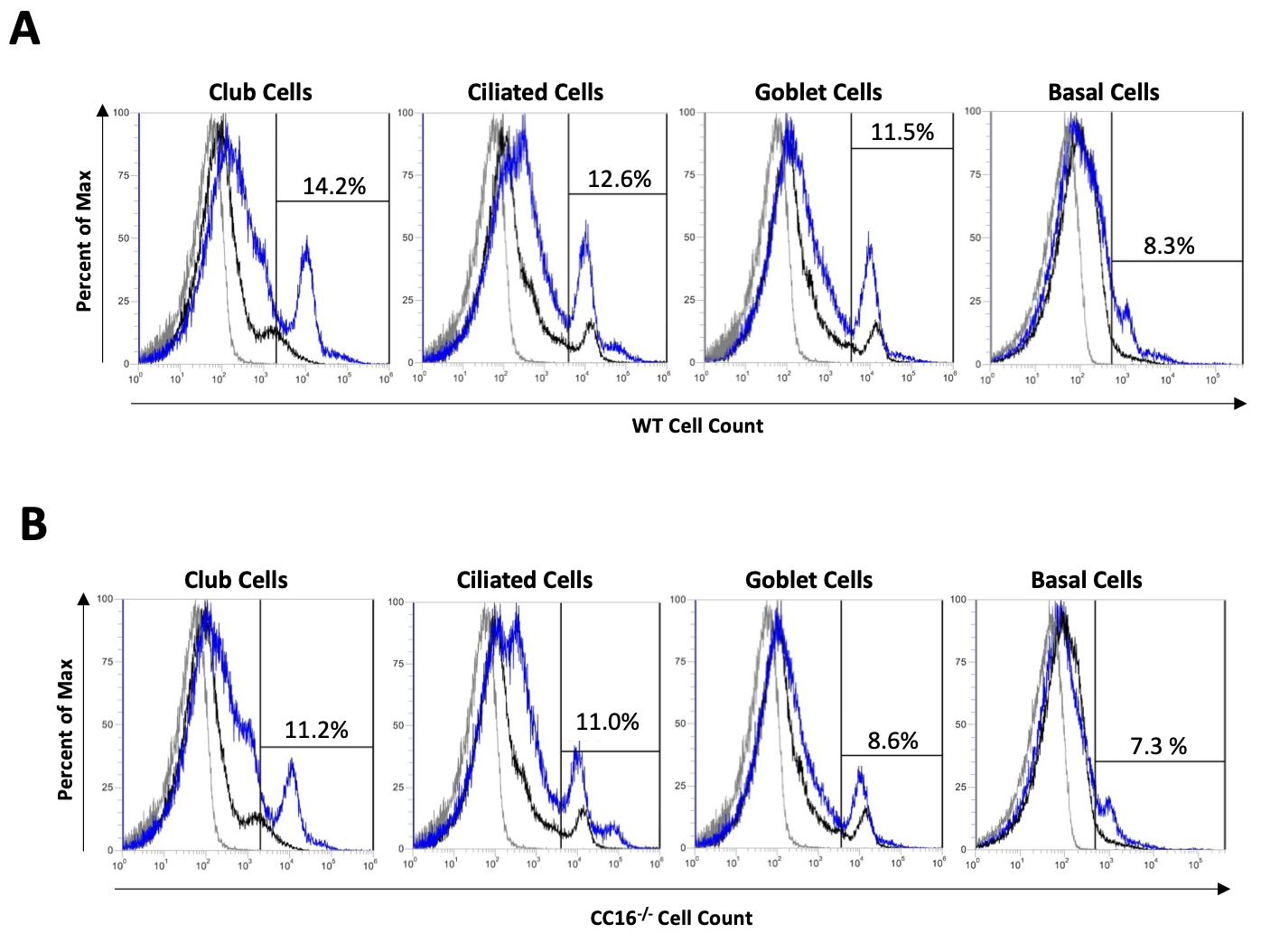

Supplement: Supplementary Figure 3 — Representative major epithelial cell populations in WT and CC16-/- mouse lungs by flow cytometry. Club (CYP2F2), ciliated (TUBA1A), goblet (MUC5AC), and basal (KRT5) cell percentages were measured in WT (A) and CC16-/- (B) mouse lungs by flow cytometry. Alexa Flour 647 was used as the secondary antibody for detection of club, ciliated, and goblet cells. Alexa Fluor 488 was used as the secondary antibody for detection of basal cells. Gray histograms represent unstained cells; black histograms represent isotype controls; and blue histograms represent experimental antibody. [file Image_3.tiff]

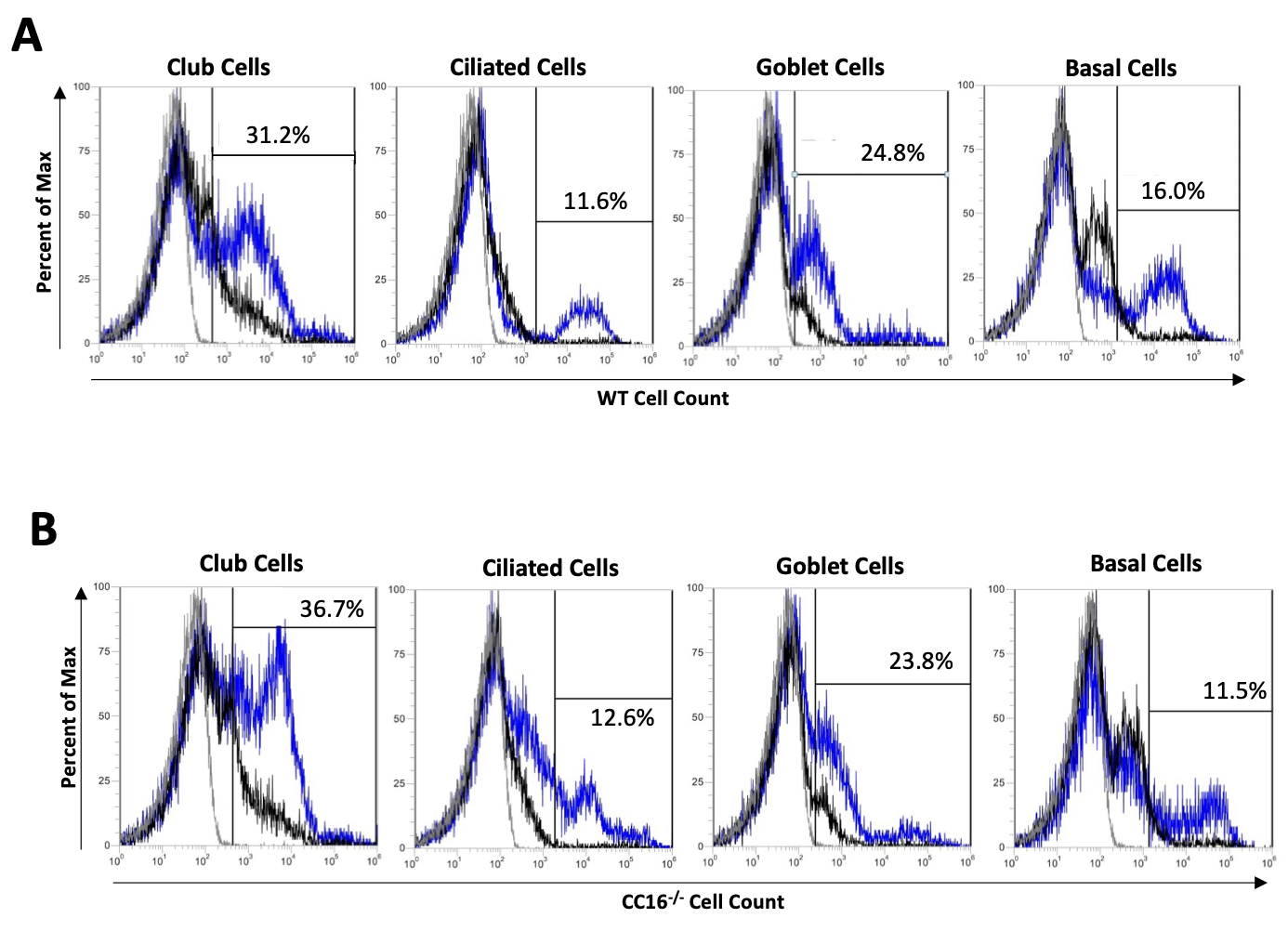

Supplement: Supplementary Figure 4 — Representative major epithelial cell populations in WT and CC16-/- MTECs by flow cytometry. Club (CYP2F2), ciliated (TUBA1A), goblet (MUC5AC), and basal (KRT5) cell percentages were measured in WT (A) and CC16-/- (B) MTECs by flow cytometry. Alexa Flour 647 was used as the secondary antibody for detection of club, ciliated, and goblet cells. Alexa Fluor 488 was used as the secondary antibody for detection of basal cells. Gray histograms represent unstained cells; black histograms represent isotype controls; and blue histograms represent experimental antibody. [file Image_4.tiff]

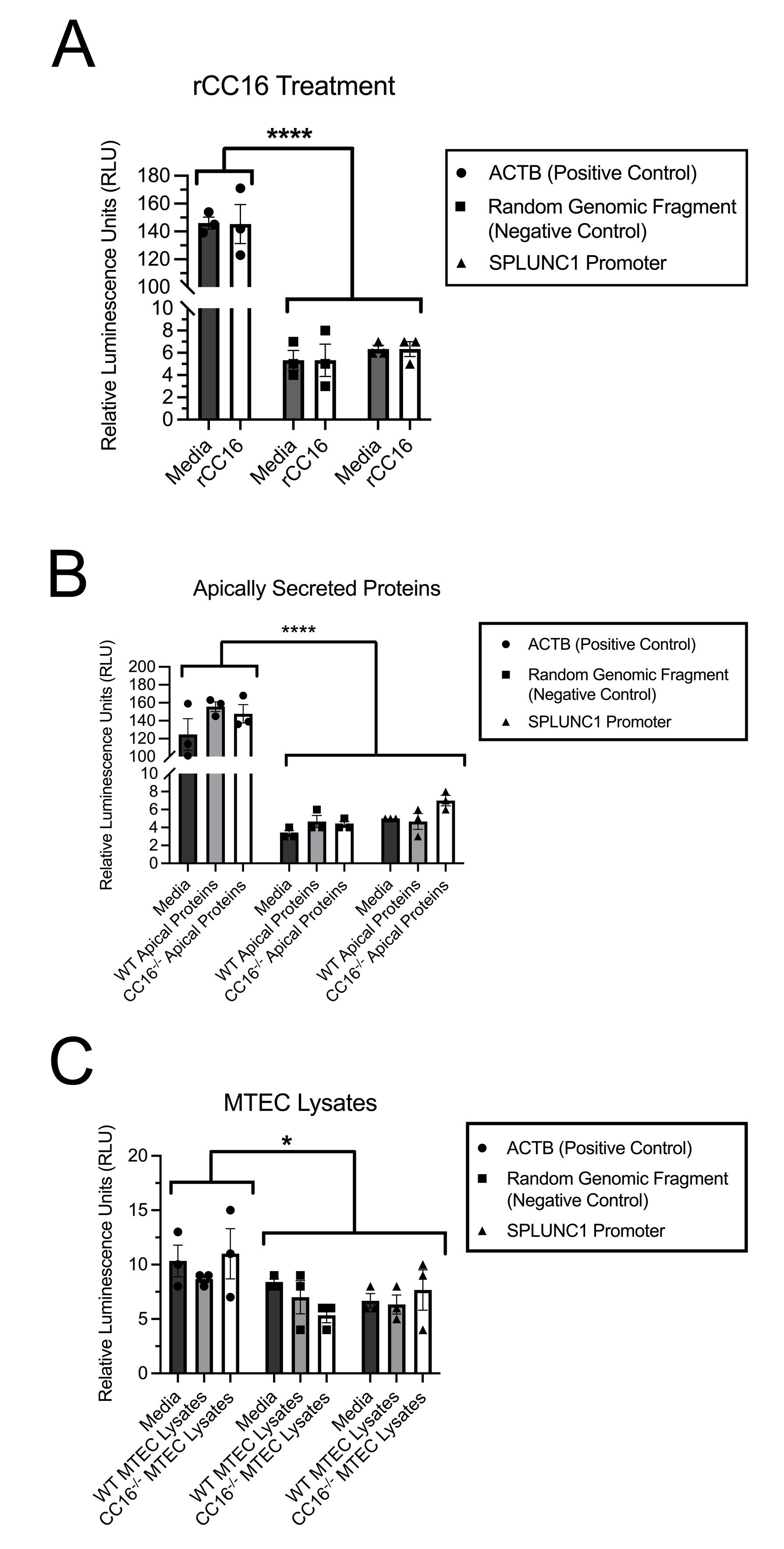

Supplement: Supplementary Figure 5 — CC16 does not directly activate the SPLUNC1 promoter. NCI-H292 were transfected with a SPLUNC1 promoter reporter construct containing a novel luminescent reporter gene (RenSP luciferase). After transfection (24 hrs), media or rCC16 (A), apically secreted proteins from WT and CC16-/- MTECs (B), and WT and CC16-/- MTEC lysates (C) were added to the transfected NCI-H292 cells (24 hrs), after which promoter activity was measured by luminescence. An actin beta (ACTB) promoter vector was used as a positive control; a random genomic fragment promoter vector was used as a negative control; and the SPLUNC1 promoter vector was used as the experimental condition. ****P<0.0001 by Two-Way ANOVA Šidák’s multiple comparison test. Data are presented as mean±SEM. [file Image_5.tif]

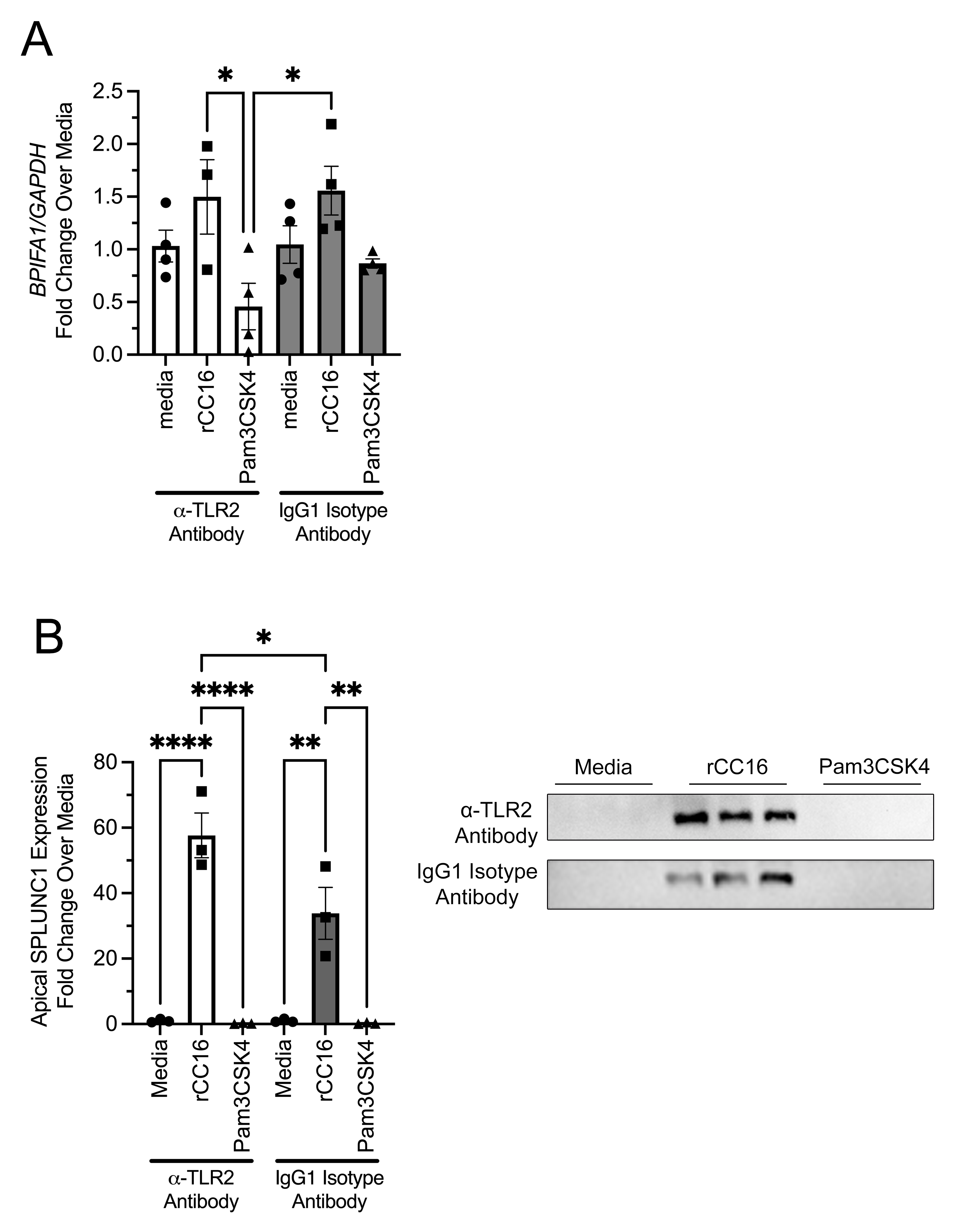

Supplement: Supplementary Figure 6 — CC16 activation of SPLUNC1 expression is independent of TLR2. (A) WT MTECs (n=4; except α-TLR2+rCC16 n=3) were treated with either a TLR2 blocking antibody (α-TLR2) or an isotype control antibody (IgG1) (10 μg/mL) for 30 min, followed by treatment with media only, rCC16 (25 μg/mL), or Pam3CSK4 (1 μg/mL) for 24 hrs. Following the treatments, Splunc1 gene expression was measured by RT-PCR with Gapdh as a housekeeping control. *P<0.05 by One-Way ANOVA Tukey’s multiple comparison test. (B) SPLUNC1 apical protein expression was assessed for media-, rCC16-, and Pam3CSK4-treated WT MTECs following incubation with an α-TLR2 antibody or IgG1 isotype antibody (10 μg/mL) by western blotting (n=3 per group). The same volume of protein (20μl) was loaded for each sample. **P<0.01, ****P<0.0001 by One-Way ANOVA Tukey’s multiple comparison test. [file Image_6.tif]
