# Supplementary material for: CC16 drives VLA-2-dependent SPLUNC1 expression
Source: Front Immunol. 2023 Nov 20;14:1277582. doi: 10.3389/fimmu.2023.1277582 (PMC10694244; doi:10.3389/fimmu.2023.1277582)
Supplement: Supplementary file 8 [file Table_1.docx]

| SARP | Cross-Sectional Cohort (Microarray mRNA) | | | Longitudinal Cohort (RNAseq) | | |
| --- | --- | --- | --- | --- | --- | --- |
|  | **All Participants  (n = 155)** | **Patients with  Asthma (n = 128)** | **Non-Asthma  Participants (n = 27)** | **All Participants  (n = 156)** | **Patients with Asthma (n = 114)** | **Non-Asthma  Participants (n = 42)** |
| Age at enrollment (years) | 37 ± 13 | 37 ± 13 | 33 ± 13 | 41 ± 13 | 41 ± 13 | 41 ± 13 |
| Sex, n (%) Female | 101 (65) | 86 (67) | 15 (56) | 99 (63) | 74 (65) | 25 (60) |
| Race (% White/African American/other)* | 62/29/9 | 60/31/9 | 70/19/11 | 67/24/9 | 63/25/12 | 69/17/14 |
| Body mass index (BMI), kg/m^2^ | 30 ± 6.8 | 30 ± 6.9 | 26 ± 5.2 | 30 ± 8.1 | 31 ± 8.7 | 28 ± 5.5 |
| Baseline Pre-BD FEV_1_ % predicted | 76 ± 22 | 72 ± 22 | 94 ± 9.1 | 82 ± 21 | 76 ± 19 | 99 ± 12 |
| Baseline Pre-BD FEV_1_/FVC | 0.72 ± 0.12 | 0.70 ± 0.12 | 0.81 ± 0.04 | 0.73 ± 0.10 | 0.70 ± 0.08 | 0.81 ± 0.04 |
